# Designed ferritin nanocages displaying trimeric TRAIL and tumor-targeting peptides confer superior anti-tumor efficacy

**DOI:** 10.1038/s41598-020-77095-x

**Published:** 2020-11-17

**Authors:** Jae Do Yoo, Sang Mun Bae, Junyoung Seo, In Seon Jeon, Sri Murugan Poongkavithai Vadevoo, Sang-Yeob Kim, In-San Kim, Byungheon Lee, Soyoun Kim

**Affiliations:** 1 Department of Biochemistry and Cell Biology, Cell and Matrix Research Institute, School of Medicine, Kyungpook National University, Daegu, 41944 Republic of Korea; 2 PrismCDX, Inc., 593-16, Dongtangiheung-ro, Hwaseong-si, Gyeonggi-do, 18469 Republic of Korea; 3 Asan Institute for Life Sciences, Asan Medical Center, University of Ulsan College of Medicine, Seoul, 138-736 Republic of Korea; 4 Department of Convergence Medicine, University of Ulsan College of Medicine, Seoul, 05505 Republic of Korea; 5 Biomedical Research Institute, Korea Institute of Science and Technology, Seoul, 02792 Republic of Korea; 6 KU-KIST Graduate School of Converging Science and Technology, Korea University, Seoul, 02841 Republic of Korea

**Keywords:** Cancer, Drug development, Nanotechnology in cancer

## Abstract

TRAIL is considered a promising target for cancer therapy because it mediates activation of the extrinsic apoptosis pathway in a tumor-specific manner by binding to and trimerizing its functional receptors, DR4 or DR5. Although recombinant human TRAIL has shown high potency and specificity for killing cancer cells in preclinical studies, it has failed in multiple clinical trials for several reasons, including a very short half-life mainly caused by instability of the monomeric form of TRAIL and rapid renal clearance of the off-targeted TRAIL. To overcome such obstacles, we developed a TRAIL-active trimer nanocage (TRAIL-ATNC) that presents the TRAIL ligand in its trimer-like conformation by connecting it to a triple helix sequence that links to the threefold axis of the ferritin nanocage. We also ligated the tumor-targeting peptide, IL4rP, to TRAIL-ATNC to enhance tumor targeting. The developed TRAIL-ATNC^IL4rP^ showed enhanced agonistic activity compared with monomeric TRAIL. The in vivo serum half-life of TRAIL-ATNC^IL4rP^ was ~ 16-times longer than that of native TRAIL. As a consequence of these properties, TRAIL-ATNC^IL4rP^ exhibited efficacy as an anti-tumor agent in vivo against xenograft breast cancer as well as orthotopic pancreatic cancer models, highlighting the promise of this system for development as novel therapeutics against cancer.

## Introduction

TRAIL (tumor necrosis factor [TNF]-related apoptosis-inducing ligand) is a promising anti-cancer agent because it is able to induce apoptosis in various types of cancer cells while it spares normal cells^1,2^. TRAIL is part of the TNF superfamily and shares a structural feature with other Type II membrane proteins^3^, namely an extracellular region that can be released as a soluble molecule and stabilized into a homotrimer through an internal zinc atom^4^. Five receptors for TRAIL have been identified: TRAIL receptor 1 (death receptor 4 [DR4]), TRAIL receptor 2 (death receptor 5 [DR5]), TRAIL receptor 3 (decoy receptor 1 [DcR1]), TRAIL receptor 4 (decoy receptor 2 [DcR2]), and the soluble receptor, osteoprotegerin (OPG)^5–7^. The death receptors, DR4 and DR5, which are overexpressed in many types of cancers, have a cytoplasmic death domain that transduces an apoptotic signal and triggers programmed cell death^8^. By contrast, the decoy receptors, DcR1 and DcR2, which have a truncated or no death domain, are unable to initiate apoptotic cell death and act as decoys^9^. OPG is another decoy receptor that binds TRAIL with low affinity, which is an interaction that appears to be of minimal physiological significance^10^. TRAIL selectively causes apoptosis in cancer cells because normal cells show an upregulation of decoy receptors in response to TRAIL and thus prevent apoptosis. Another benefit of directly targeting DRs is that it is targeting an apoptotic pathway that does not involve the p53 tumor-suppressor protein, which is the target of many conventional cancer drugs but is inactivated in more than half of human cancers^11,12^. Notably, pancreatic tumor cells mostly do not respond to conventional radio- and chemotherapy, mainly due to p53 mutations. Pancreatic ductal adenocarcinoma (PDAC) thus remains the fourth leading cause of cancer-related death worldwide, and the development of novel therapeutic approaches for treating this disease is urgently needed.

A number of studies have intensely investigated TRAIL in anticipation of clinical trials. However, translation of TRAIL into the clinic has been confounded by its short half-life, inadequate delivery methods, and TRAIL-resistant cancer cell populations^13,14^. Even after delivery into the tumor, TRAIL activity is evaded by TRAIL-resistant cancer cells, which express decoy receptors or inhibitors of the apoptotic pathway, such as cellular FADD-like interleukin-1β-converting enzyme (FLICE)-inhibitory protein (c-FLIP)^15^. Combining TRAIL with established chemo- or radiotherapy might overcome the resistance of some tumor cells to therapy. To date, however, the only recombinant human TRAIL-related product approved for clinical use is a monomeric form of TRAIL, named dulanermin (amino acids 114–281 of TRAIL) in combination with chemotherapeutics^14^. However, this monomeric TRAIL has failed to show significantly improved anti-tumor effects in clinical trials compared with standard therapy^13,16^. The serum half-life of dulanermin in humans is only 30–60 min, possibly the primary reason for its modest efficacy^17^. The half-life of TRAIL in animal models is even shorter: 3–5 min in rodents and 24–31 min in cynomolgus monkeys and chimpanzees. This short half-life is attributable to the unstable character of the monomeric form of TRAIL protein and its rapid clearance via the kidneys^18^. To prolong the half-life of active TRAIL, researchers have made numerous attempts to improve TRAIL stability and prepare trimeric formulations of recombinant TRAIL, including FLAG-tag-mediated crosslinking^19^; fusion of trimerization domains, for example using a leucine zipper^20^ or trimeric coiled-coil domain^21^; conjugation to various nanoparticles^22–24^; or fusion to circulating cells^25,26^. However none of these approaches has yielded an effective anti-cancer therapy for human patients, despite the robust cytotoxic effects obtained in vitro and in vivo^14,27^. Other potential problems hindering the therapeutic efficacy of TRAIL may arise from the fact that active recombinant TRAIL might be diluted through binding to decoy receptors in normal tissues^28^.

Nanoparticle-based technology is an important and growing area of cancer therapeutics, reflecting the ability of nanoparticles—because of their nanometer scale—to enhance the circulation of their payloads, such as chemical drugs and protein ligands, and cause passive accumulation of nanoparticles in tumor sites through the enhanced permeability and retention (EPR) effect^29–31^. Among nanoparticles, naturally produced protein-based nanoparticles offer advantages of biostability, biocompatibility, and biodegradability compared with synthetic polymers; they also can be manipulated for various applications owing to their perfect and complex symmetric assembly^32,33^. The ferritin nanocage is a well-studied biological nanoparticle that shows the capacity to sequester a variety of minerals and metals within its cage structure. Ferritin has been employed in various medical-related applications, including as contrast agents for medical imaging^34–36^, targeted drug delivery^37–39^, vaccine development^40–42^, and as diagnostic/therapeutic nanoparticle platforms^43–46^. The ferritin nanocage is assembled from 24 monomer subunits through 2-, 3-, and fourfold symmetry, thus allowing for the loading of ligands into the designated interfaces with the expected arrangement^47–49^. For example, hemagglutinin has been inserted at the threefold interface of adjacent subunits such that it assembles to generate eight trimeric viral spikes on the ferritin surface, yielding a potent influenza vaccine^41^.

Here, we describe a trimer delivery platform using the ferritin protein nanocage, termed active trimer nanocage (ATNC), that overcomes the drawbacks of TRAIL as an anti-cancer drug in the clinical realm in two ways. First, linking the triple helix sequence moiety found in human pulmonary surfactant-associated protein D^21^ to the exposed threefold axis of ferritin nanocages assists in formation of a trimer-like structure of the connected TRAIL. Second, multi-display of tumor-targeting IL4 receptor-binding peptides (IL4rPs) on the surface of the ATNC overcomes the mistargeting of TRAIL caused by dilution through binding to decoy receptors or rapid elimination from the circulation by the kidneys. IL4 receptor (IL4R) is a biomarker for tumor cells including breast cancer and lung cancer^50,51^. IL4R binding peptide (IL4rP), homologous to the sequence of IL4, was identified via screening of a phage-displayed peptide library^52^. IL4rP was shown to specifically bind to IL4R and selectively target IL4R-expressing tumors^53^. This targeted TRAIL-ATNC (TRAIL-ATNC^IL4rP^) showed enhanced affinity, stability, and excellent apoptotic activity in vitro, as well as tumor targeting and enhanced anti-tumor efficacy in animal tumor model. This active trimer-delivery platform provides a scaffold for presenting trimeric proteins such as TNF-α family ligands or their receptors and promises possible therapeutic applications of various ATNCs in different diseases.

## Results

### Design of active trimer ferritin nanocages

**Figure 1:**
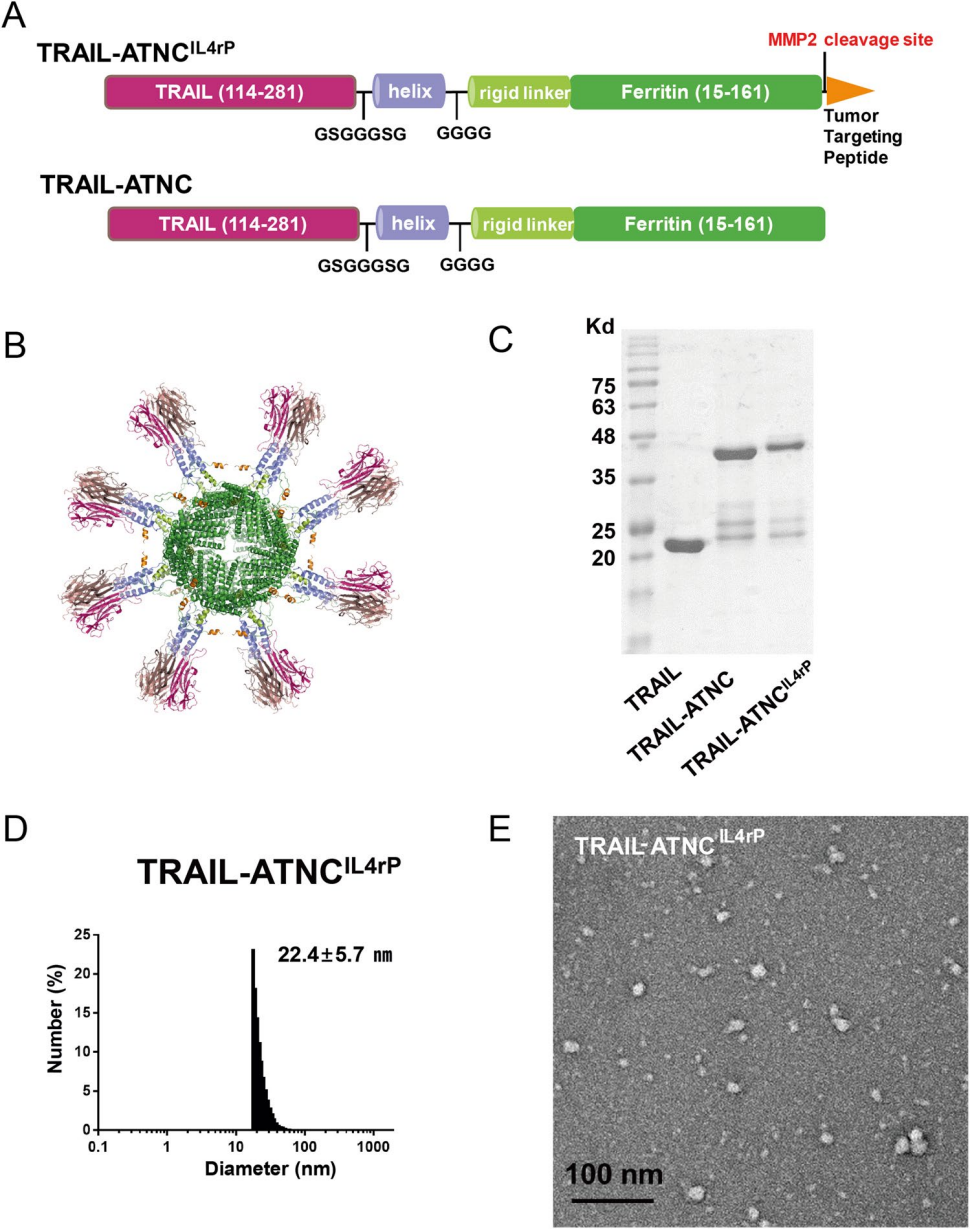
Design and physicochemical characterization of TRAIL-Active Trimer Nanocage (ATNC). (**A**) Protein primary structure diagram of TRAIL-ATNC and TRAIL-ATNC^IL4rP^. The C terminus of the ecto-domain of TRAIL (114–281) was fused to the N-terminus of the short version of the human ferritin subunit (15–161) by a helix and rigid linker. The IL4 receptor binding peptide (IL4rP: CRKRLDRNC) was inserted into the C-terminal of the ferritin as a tumor targeting peptide. A matrix metalloproteinase-2 (MMP2) cleavage site (GPLGLAG) was inserted between the IL4rP and the ferritin. (**B**) 3D picture of the TRAIL-ATNC^IL4rP^ with eight TRAIL homotrimers displayed on the surface of ferritin nanocages. The image was drawn using PyMOL v0.99 (The PyMOL Molecular Graphics System, Schrödinger, LLC). (**C**) SDS-PAGE of the purified TRAIL-ATNC and TRAIL-ATNC^IL4rP^ proteins. The major bands correspond with the expected sized of TRAIL-ATNC (44.1 kDa) and TRAIL-ATNC^IL4rP^ (46.7 kDa). (**D**) DLS analysis of TRAIL-ATNC^IL4rP^. (**E**) Transmission electron microscopy image of TRAIL-ATNC^IL4rP^.

Ferritin forms a cage-like supramolecular assembly composed of 24 subunits arranged with a 4–3-2 symmetry structure around a hollow interior. Based on a structural analysis of *Helicobacter pylori* ferritin, Kenekiyo et al. developed a nanoparticle vaccine that mimics the trimeric influenza viral spike by inserting influenza virus hemagglutinin (HA) into the aspartic acid (Asp) at residue 5 near the N-terminus, exposed on the threefold axis of ferritin^41^. A sequence comparison of human ferritin heavy chain (FtH) and *H. pylori* ferritin showed that Asp15 of human FtH is aligned with Asp5 of *H. pylori* ferritin (sFig. 1A). Asp15 of human FtH, the starting residue of helix I, is readily solvent accessible, and the distance between each Asp15 on the threefold axis is 28 Å. When the TRAIL trimer was stabilized by Zn^2+^, the N- and C- termini are positioned triangularly at distances of 23 and 11 Å, respectively (sFig. 1B). Trimer formation was aided by ligating the triple helical domain of pulmonary surfactant-associated protein D to the C-terminus of TRAIL (sFig. 1C). Various constructs of the TRAIL-ferritin fusion platform, with full length (amino acids 15–183) or short versions of ferritin (amino acids 15–161) and with flexible or rigid linkers, were assessed for their cytotoxic activity against cancer cells (sFig. 2A–D). Against our expectations, the TRAIL-GS-helix or other TRAIL-GS-helix-conjugated ferritin constructs were not efficient in killing cancer cells compared to the monomeric form of TRAIL. We hypothesized that this inefficiency is because the C-terminal triangle of TRAIL is buried behind the N-terminal triangle, which makes proper conformation of a TRAIL-trimer difficult (sFig. 1B). To address this problem, we added flexible linkers consisting of small amino acids (GSGGGSG) that could form a bridge between the C-terminal of TRAIL and the triple helix, which resulted in significantly improved cytotoxic activity (sFig. 2A and D). This modified TRAIL-conjugated ferritin was called TRAIL-ATNC and showed cytotoxic activity ~ 10 times greater than monomeric TRAIL or TRAIL-_GGGSG_-helix trimer (sFig. 2E). We employed a short version of ferritin (amino acids 15–161) that lacks the N-terminal flexible segment (residues 1–14) and the short fifth α-helix (helix V) as a scaffold due to its improved cytotoxic activity. In the intact ferritin complex, helix Vs are located in the interior space of the cage and are not critically involved in cage formation^54^. Our previous studies demonstrated that ferritin without helix V forms a cage structure, is expressed with higher yield in *Escherichia coli* than wild-type ferritin, and exposes N- and C-terminal fusion payloads on the surface in a more accessible fashion than wild-type ferritin^55^. The TRAIL-ATNC construct and the expected trimer display are depicted in Fig. 1A. IL4rP (CRKRLDRNC)^43^ was ligated to the C-terminal end as a tumor-targeting peptide moiety, and a matrix metalloproteinase-2 (MMP2) cleavage site (GPLGLAG)^55^ was inserted in the middle of the flexible linker between IL4rP and the ferritin carrier to prevent unwanted IL4 receptor-mediated endocytosis after targeting. Upon assembly of the 24 monomeric ferritin subunits into the cage structure, eight TRAIL homotrimers are displayed on the surface of ferritin nanocages (Fig. 1B). Sequence information of TRAIL-ATNC with or without IL4rP peptide is shown in sFig. 3 and 4. Exposure of the functional IL4rP on the surface of the TRAIL-ATNC^IL4rP^ was verified by its specific binding to IL4 receptor using SPR analysis (sFig. 5).

### Biosynthesis and physicochemical characterization of TRAIL-ATNC

TRAIL-ATNCs with or without IL4rP were successfully expressed and purified as recombinant proteins in soluble form in *E. coli* (Fig. 1C). To identify the co-purified bands around 25 kDa, the purified TRAIL-ATNCs were analyzed by mass spectroscopy (LC-ESI Q-TOF) and western blot (sFig. 6A and B). The mass analysis of TRAIL-ATNC and TRAIL-ATNC^IL4rP^ showed identical sized peaks (MW = 23,899.0 and 23,898.4, respectively) around 25 kDa. The molecular weight of this peak is best matched with a TRAIL-containing fragment of amino acids 18–228 (MW = 23,896.65) as shown in the schematic of sFig. 6A. The western blot showed that one of the 25 kDa bands was stained by anti-TRAIL Ab, suggesting that the TRAIL-containing fragments are partially cleaved out from both TRAIL-ATNC and TRAIL-ATNC^IL4rP^ during purification.

Cage formation by purified TRAIL-ATNCs was evaluated by dynamic light scattering (DLS) and transmission electron microscopy (TEM) (Fig. 1D,E). The TRAIL-ATNC with IL4rP formed nano-size particles with an average diameter of 22.4 nm as measured by DLS. TEM images revealed a spherical, but roughly shaped, particle architecture with an average size of 19.41 ± 3.14 nm. The TRAIL-ATNC without IL4rP also formed nano-sized particles revealed by DLS and TEM (sFig. 7A and B) but the average size by DLS (41.0 nm) or TEM (24.32 ± 5.36 nm) was larger than that of TRAIL-ATNC^IL4rP^. The larger size by DLS than by TEM, likely reflects solvation of the sample detected for DLS measurements^56^. Minor heterogeneous particles were observed in TEM images from TRAIL-ATNC with or without IL4rP and also in the low magnification TEM images of TRAIL-ATNCs, implying the presence of a population of improperly folded complexes (sFig. 8). However, the rounder shape of TRAIL-ATNC with IL4rP compared to its heterogeneous particles suggests that there is a fair amount of properly folded complexes that accommodate TRAIL. The TRAIL-ATNCs were further analyzed using the Multiangle Light Scattering (MLS) (sFig. 9), indicating that TRAIL-ATNC and TRAIL-ATNC^IL4rP^ did form 24-mer cages. Estimated molecular weights of TRAIL-ATNC (798.8 kDa) and TRAIL-ATNC^IL4rP^ (640.2 kDa) were larger than that of the ferritin cage assembled by 24 short ferritin monomers (MW 410 kDa). Smaller sized peaks were observed, which were likely monomer, dimer, trimer, and hexamer conformations. Particle aggregation was specifically visible in TEM images of TRAIL-ATNC, and aggregated particles eluted in void fraction seemed to form more in TRAIL-ATNC than in TRAIL-ATNC^IL4rP^. Collectively, however, these observations suggest that TRAIL-ATNCs that present multimeric TRAILs on nanocages were successfully generated.

### In vitro pro-apoptotic activity of TRAIL-ATNCs

To verify that TRAIL-ATNC targets the TRAIL receptor on the surface of tumor cells and mediates programed cell death, we first examined the expression of the TRAIL death receptors, DR4 and DR5, in A549 and H1703 human lung cancer cells, and MDA-MB-231 human breast cancer cells (sFig. 10A-C), all of which are known to express higher amounts of DR4/DR5 than DcR1/DcR2^57,58^. Consistent with previous reports, we found that, compared with IgG controls, DR4 and DR5 levels were more than fivefold higher in MDA-MB-231 and A549 cells, and twofold higher in H1703 cells, whereas the levels of DcR1 and DcR2 were similar to those in controls. Both H1703 and MDA-MB-231 cells showed high susceptibility to TRAIL-mediated death, but A549 cells were unaffected by exposure to TRAIL owing to expression of c-FLIP, an inhibitor of the apoptotic pathway^59^. Based on TRAIL receptor expression and susceptibility, we chose MDA-MB-231 cells to verify TRAIL-ATNC cytotoxicity. IL4 receptor (IL4R) expression was also confirmed in MDA-MD-231 cells to achieve IL4R dependent targeting by TRAIL-ATNC^IL4rP^ (sFig. 10D).

**Figure 2:**
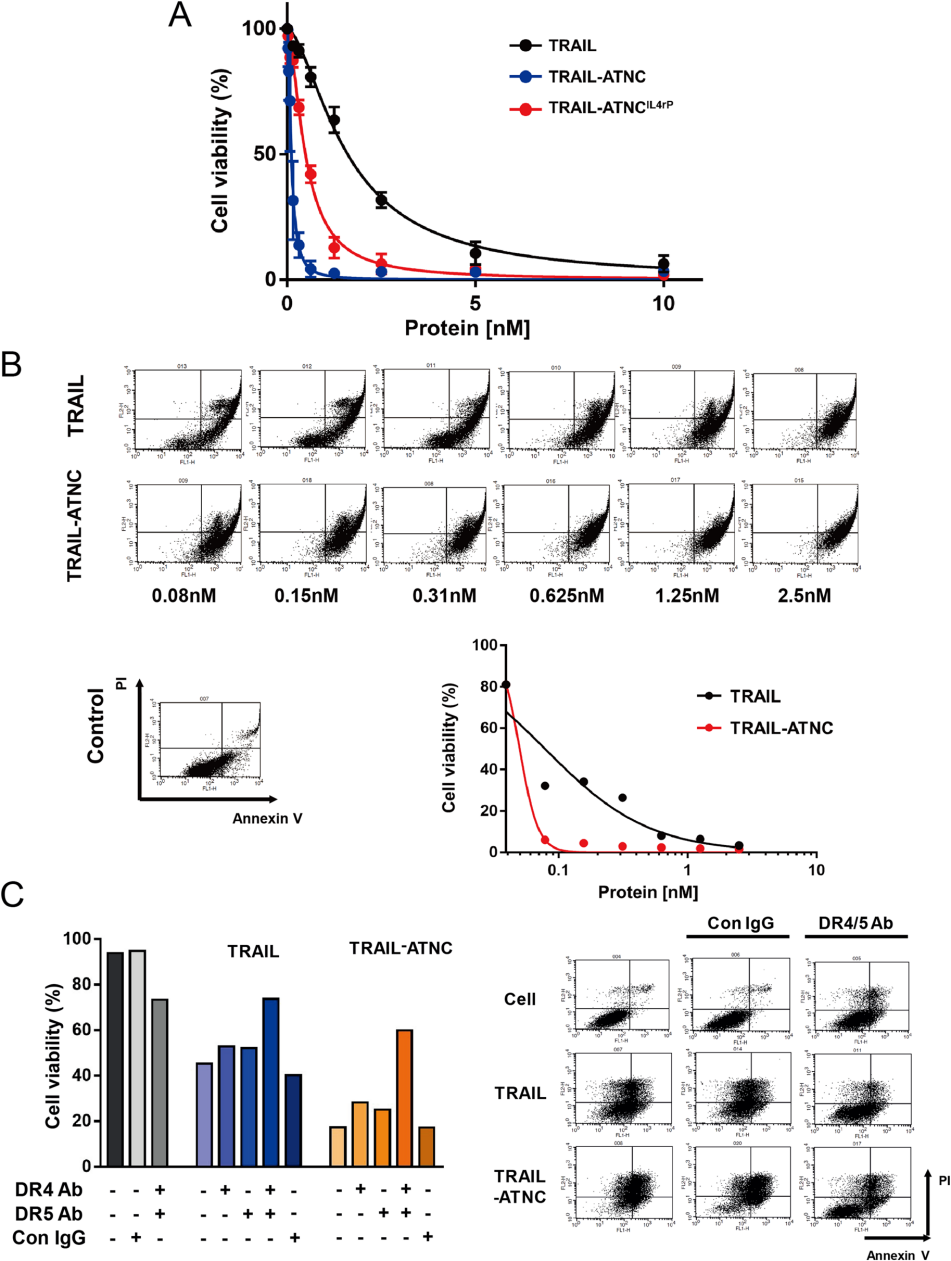
Cancer cell cytotoxicity of TRAIL-ATNCs via TRAIL receptors. (**A**) Cell viability following treatment with TRAIL, TRAIL-ATNC, or TRAIL-ATNC^IL4rP^. MDA-MB-231 cells were incubated with proteins for 24 h and then measured by EZ-count assay. Data represent means ± SEM. (**B**) The apoptosis inducing ability of TRAIL or TRAIL-ATNC was analyzed with Annexin V and propidium iodide (PI) double staining using flow cytometry. The percentage of annexin V/PI double-negative (viable) cells was plotted. (**C**) Apoptosis inducing ability of TRAIL or TRAIL-ATNC was blocked by anti-DR4/DR5 antibodies. MDA-MB-231 cells were pre-incubated with DR4/DR5 blocking antibody or control IgG for 1 h and were incubated with TRAIL or TRAIL-ATNC for 3 h. Cell viability was measured with Annexin V and PI double staining using flow cytometry.

To assess the extent of TRAIL-mediated apoptosis of TRAIL-ATNCs with or without IL4rP, we first compared the viability of MDA-MB-231 cells that were treated with a series of concentrations of TRAIL-ATNC, TRAIL-ATNC^IL4rP^, and monomeric TRAIL (Fig. 2A). TRAIL induced the least cell death, while TRAIL-sensitive MDA-MB-231 cells treated with the TRAIL-ATNCs showed a larger increase in cell death that was concentration-dependent. Specifically, TRAIL-ATNC exhibited a 50% inhibitory concentration (IC_50_) value of 0.11 nM in MDA-MB-231 cells and TRAIL-ATNC^IL4rP^ showed an IC_50_ value of 0.48 nM; the IC_50_ value for TRAIL in comparison was 1.6 nM. To determine whether the observed TRAIL-ATNC–induced tumor cell death reflected a programmed cell death process, cell apoptosis was analyzed using annexin V/propidium iodide (PI) double staining in conjunction with fluorescence-activated cell sorting (FACS) analysis (Fig. 2B). Apoptotic and necrotic cell death were indicated in MDA-MB-231 cells treated with TRAIL-ATNCs as a concentration-dependent increase in the percentage of annexin V/PI-positive cells was induced (annexin V-positive: early apoptosis; PI-positive: late apoptosis and necrosis), further confirming that the observed apoptosis of MDA-MB-231 cells was induced by TRAIL-ATNCs. Remarkably, a concentration as low as 0.08 nM of TRAIL-ATNCs was sufficient to cause a 98% increase in apoptotic annexin V-positive cells, whereas a nearly eightfold higher concentration of TRAIL (0.625 nM) was required to produce a comparable increase in apoptotic cells (Fig. 2B). Furthermore, concurrent pre-incubation with anti-DR4 and anti-DR5 antibodies significantly rescued the viability of MDA-MB-231 cells treated with ~ 80% cytotoxic concentrations of TRAIL (2.5 nM) or TRAIL-ATNCs (0.3125 nM) (Fig. 2C). Single treatment with either antibody alone was ineffective. These results confirm that TRAIL-ATNCs mediate cell death specifically via TRAIL-receptors—both DR4 and DR5—on the surface of tumor cells.

### Binding kinetics and affinity of TRAIL-ATNCs

**Figure 3:**
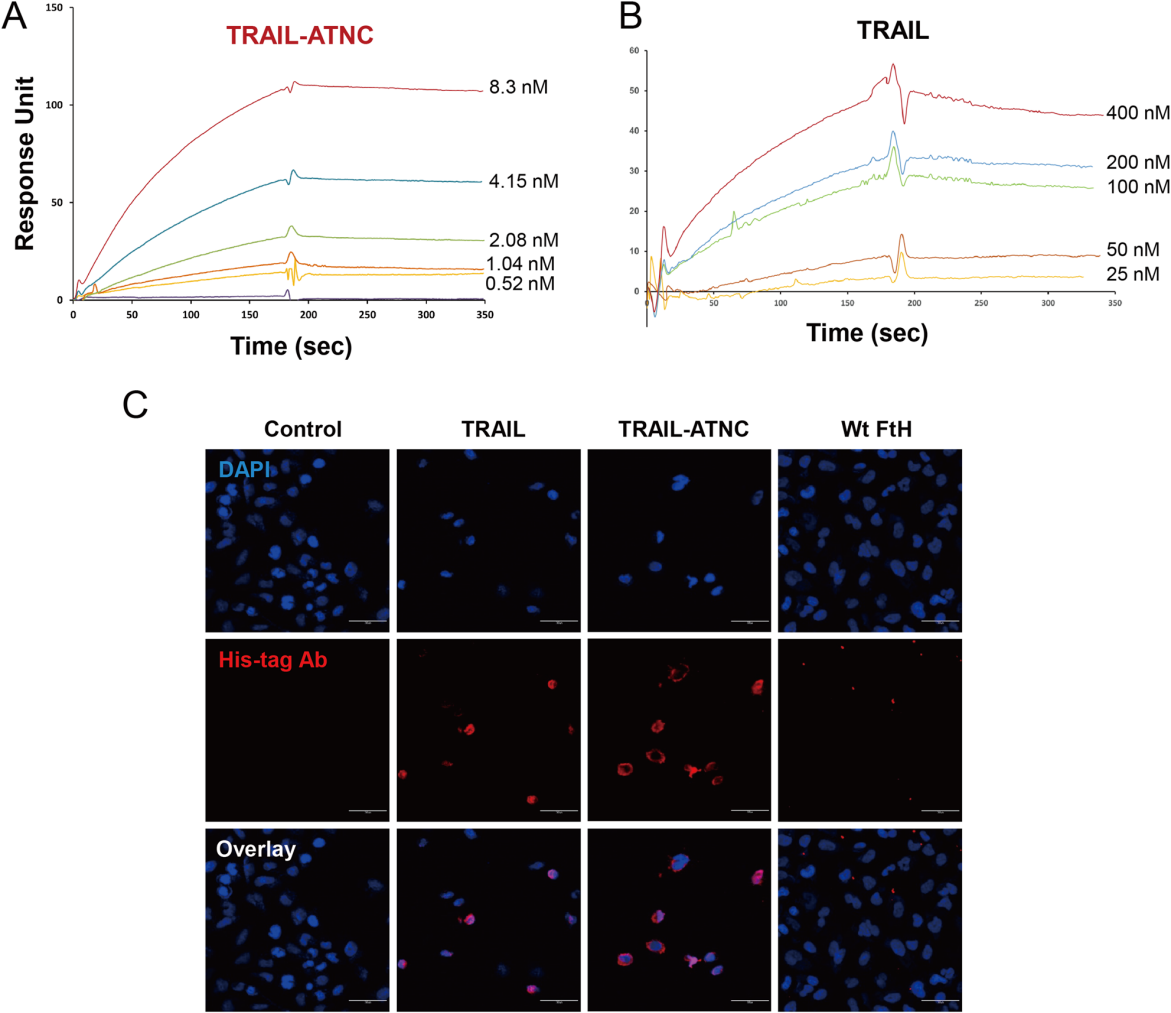
Binding kinetics of TRAIL-ATNC with TRAIL receptor. (**A**,**B**) Surface plasmon resonance analysis for the binding kinetics of TRAIL and TRAIL-ATNC with TRAIL receptor (DR5). The concentrations of TRAIL (25–400 nM) and TRAIL-ATNC (0.52–8.33 nM) were injected to DR5 coated dextran-coated chips. (**C**) Representative fluorescence images of TRAIL, TRAIL-ATNC, and wild type ferritin (Wt FtH) bound to A549 cells, showing efficient cell binding of TRAIL and TRAIL-ATNC. A549 cells were treated with 50 nM of each protein for 1 h at 4 ℃ and immunostained with anti-His tag antibodies (red). Nuclei were counterstained with DAPI (blue). Scale bars: 30 µm.

To examine the binding kinetics and affinity of TRAIL-ATNCs, we immobilized an extracellular region of the DR5, applied a series of different concentrations of TRAIL-ATNC or TRAIL, and compared binding kinetics using surface plasmon resonance (SPR) analysis. Monomeric TRAIL bound to DR5 with an affinity (K_**D**_) of 6.0 × 10^–8^ M; in comparison, TRAIL-ATNCs bound to DR5 with sub-nanomolar affinity (2.5 × 10^–10^ M)—a K_**D**_ value ~ 235-times lower than that of TRAIL (Table 1 and Fig. 3A,B). Association rates were 15-fold higher and dissociation rates were 16-fold lower for TRAIL-ATNC compared with monomeric TRAIL, indicating that the clustered structures of TRAIL on the surface of TRAIL-ATNC are readily recognized by its receptors and form a stable complex. To test whether TRAIL-ATNC targets TRAIL receptors that are naturally expressed on the cell membrane, we examined specific binding of TRAIL-ATNCs to A549 cells, which highly express TRAIL death receptors despite being resistant to TRAIL-induced apoptosis (Fig. 3C). TRAIL and TRAIL-ATNC bound to A549 cells, whereas wild-type FtH did not. Incubation with TRAIL or TRAIL-ATNC caused a moderate decrease in total cell numbers because these cells are not completely impervious to the effects of TRAIL at the concentrations used. MDA-MB-231 cells could not be employed in these assays because of the potent cytotoxicity of TRAIL and TRAIL-ATNC towards these cells. Taken together, these results demonstrate that TRAIL-ATNCs directly bind to purified DR5 with ~ 235-fold higher affinity than the monomeric form of TRAIL and mediate apoptotic cell death through specific interactions with DR4/DR5.

**Table 1:** Binding kinetics of TRAIL-ATNC and TRAIL against TRAIL receptor DR5.

Ligand	k_on_ (M^−1^ S^−1^)^a^	k_off_ (S^−1^)^a^	K_D_ (M)^b^
TRAIL-ATNC	(3.77 ± 0.24) × 10^5^	(9.15 ± 9.14) × 10^–5^	(2.56 ± 2.58) × 10^–10^
TRAIL	(2.58 ± 0.60) × 10^4^	(1.50 ± 0.38) × 10^–3^	(6.02 ± 1.99) × 10^–8^

^a^Obtained by saturated binding responses at least three independent runs of SPR measurements.

^b^K_D_ = k_off_/k_on_.

### Pharmacokinetic profile of TRAIL-ATNC with or without IL4rP versus native TRAIL in mice

**Figure 4:**
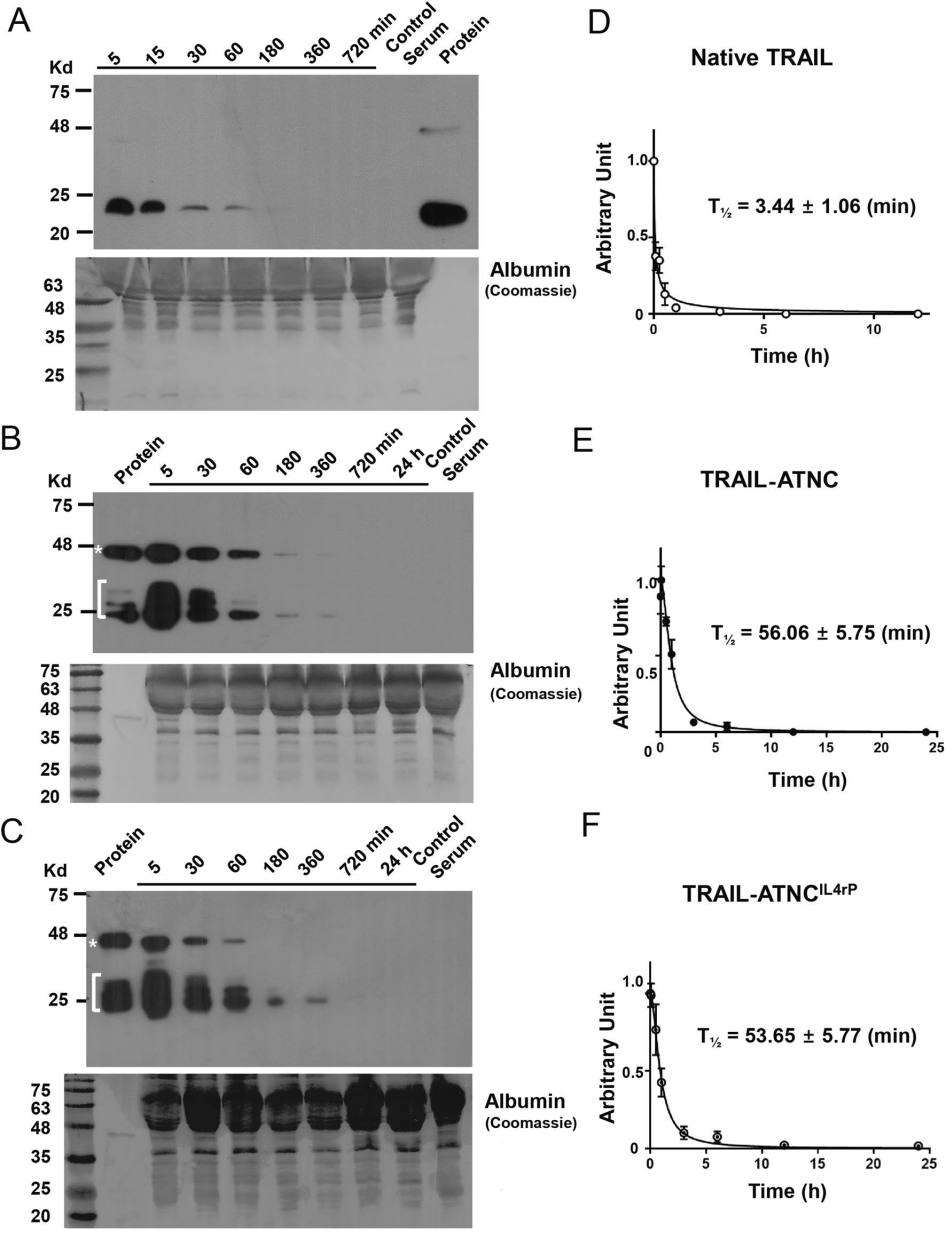
Pharmacokinetics analysis of TRAIL, TRAIL-ATNC, and TRAIL-ATNC^IL4rP^. Proteins were injected into mice using retro orbital injection (n = 3 for each protein). (**A**–**C**) Relative serum concentrations of native TRAIL (**A**), TRAIL-ATNC (**B**), and TRAIL-ATNC^IL4rP^ (**C**) were evaluated at periodic intervals by western blotting with an antibody against human TRAIL (Protein, estimated total amount of each proteins; Control serum, serum derived from the untreated mice). The quantification of the amount of TRAIL proteins present in each sample was done using Image J program. (**D**–**F**) Estimated half-lives (T_1/2_) of TRAIL (**D**), TRAIL-ATNC (**E**), and TRAIL-ATNC^IL4rP^ (**F**) were calculated using GraphPad Prism v7.0. Error bars represent ± SEM.

Next, we investigated the pharmacokinetic profiles of TRAIL-ATNC, TRAIL-ATNC^IL4rP^, and native TRAIL. To this end, mice were injected retro orbitally (r.o.) with TRAIL-ATNC, TRAIL-ATNC^IL4rP^, or native TRAIL, and relative serum concentrations of proteins were evaluated at periodic intervals by Western blot analysis (Fig. 4A–C). Instead of His-tagged TRAIL, we used native TRAIL for pharmacokinetic assays to compare with previous data obtained using native TRAIL without the His-tag. Although the cytotoxic activity of His-tagged TRAIL was similar to that of native TRAIL (sFig. 11), His-tagged TRAIL was previously shown to cause a degree of hepatotoxicity^60^. The concentrations of TRAIL-ATNC with or without IL4rP decayed in vivo at a much slower rate than that of native TRAIL. No non-specific bands were detected in serum from control mice. The half-lives of TRAIL-ATNC, TRAIL-ATNC^IL4rP^, and native TRAIL were estimated to be 56.1 ± 5.8 min, 53.6 ± 5.8 min, and 3.4 ± 1.1 min, respectively (Fig. 4D–F), representing an approximately 16-fold longer half-life for TRAIL-ATNC with or without IL4rP. The half-life value for native TRAIL in nude mice obtained here is consistent with the ~ 3–6 min half-life observed in previous studies^18,61,62^. These results indicate that TRAIL-ATNC is less rapidly eliminated and more stable than native TRAIL in vivo.

Collectively, these results suggest that TRAIL-ATNC displays native trimer-like TRAIL on a short ferritin nanocage, and improves its in vivo stability, supporting the potential of TRAIL-ATNCs as a promising apoptotic agent against tumor cells.

### Tumor homing of IL4rP-conjugated TRAIL-ATNC

**Figure 5:**
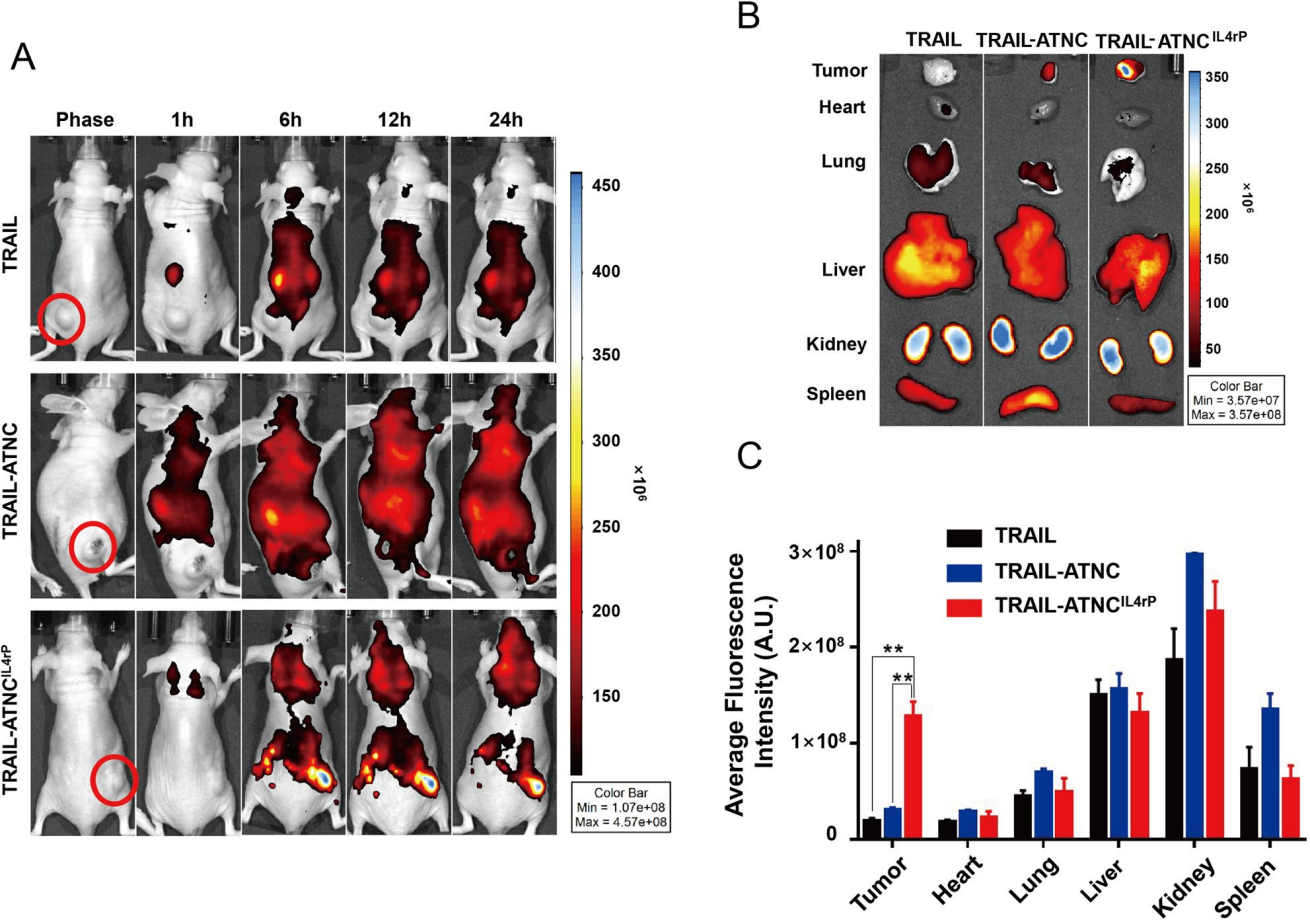
Bio-distribution of TRAIL, TRAIL-ATNC, and TRAIL-ATNC^IL4rP^. (**A**) Representative images of the bio-distribution of TRAIL, TRAIL-ATNC, and TRAIL-ATNC^IL4rP^. Mice bearing MDA-MB-231 tumor were intravenously injected with FPI774-labeled TRAIL, TRAIL-ATNC, or TRAIL-ATNC^IL4rP^ followed by in vivo scanning by IVIS imaging system. Equal amounts of fluorescence of proteins were injected. (**B**,**C**) At 24 h post-injection, mice were sacrificed and organ/tissues were collected. Ex vivo bio-distribution was examined for TRAIL, TRAIL-ATNC, and TRAIL-ATNC^IL4rP^ mice group. The average fluorescence intensity of each organ was measured (**C**). Data represent means ± SEM (**p < 0.01; T-test).

Prior to assessing the anti-tumor efficacy of TRAIL-ATNCs, we examined the efficiency of its delivery to the tumor. Previous studies showed that the homing and anti-tumor activity of TRAIL was improved by conjugation of a tumor-targeting moiety^63,64^. To examine the tumor-delivery efficiency of IL4rP-conjugated TRAIL-ATNCs (TRAIL-ATNC^IL4rP^), we intravenously injected mice bearing MDA-MB-231 tumor xenografts with FPI774-labeled TRAIL-ATNC^IL4rP^, TRAIL-ATNC or TRAIL, and then scanned mice using an IVIS imaging system at different post-injection times. All proteins were injected as preparations containing equal amounts of fluorescence so as to allow direct comparisons of tumor-targeting efficiency. Surprisingly, whole-body scans showed no significant tumor homing of TRAIL or TRAIL-ATNC; fluorescence signals in tumors were detected only in TRAIL-ATNC^IL4rP^–injected animals and persistent for 24 h (Fig. 5A). The tumor targeting of TRAIL-ATNC^IL4rP^ was further verified by ex vivo analysis of excised tumor tissues 24 h post-injection. As shown in Fig. 5B and C, the average tumor uptake of TRAIL-ATNC^IL4rP^ was approximately 4-times higher than that of TRAIL-ATNC and 6-times higher than that of TRAIL. These results indicate that tumor targeting was significantly improved by conjugation of the tumor targeting peptide, IL4rP. Minor increases in targeting of TRAIL-ATNC compared to monomeric TRAIL are likely attributable to passive delivery via the EPR effect.

The expression of a physiological receptor of ferritin, transferrin receptor 1 (TfR1), is elevated in liver and brain tissue under an inflammatory condition as well as in human cancer cells^65^. To examine the possible impact of TfR1 in the pharmacokinetics of TRAIL-ATNC, we tested whether the TRAIL-ATNC can bind to TfR1 using surface plasmon resonance (SPR) analysis (sFig. 12). TRAIL-ATNC (20 ~ 166 nM) did not bind to TfR1, while the wildtype FtH (50 nM) bound to TfR1. This is most likely because the TfR1 binding is sterically hindered by 24 TRAILs on the ferritin surface. Based on the results, it is unlikely that the ferritin receptor, TfR1, can influence the pharmacokinetics of the TRAIL conjugates.

### In vivo pro-apoptotic activity of TRAIL-ATNC^IL4rP^ in a breast cancer xenograft model

**Figure 6:**
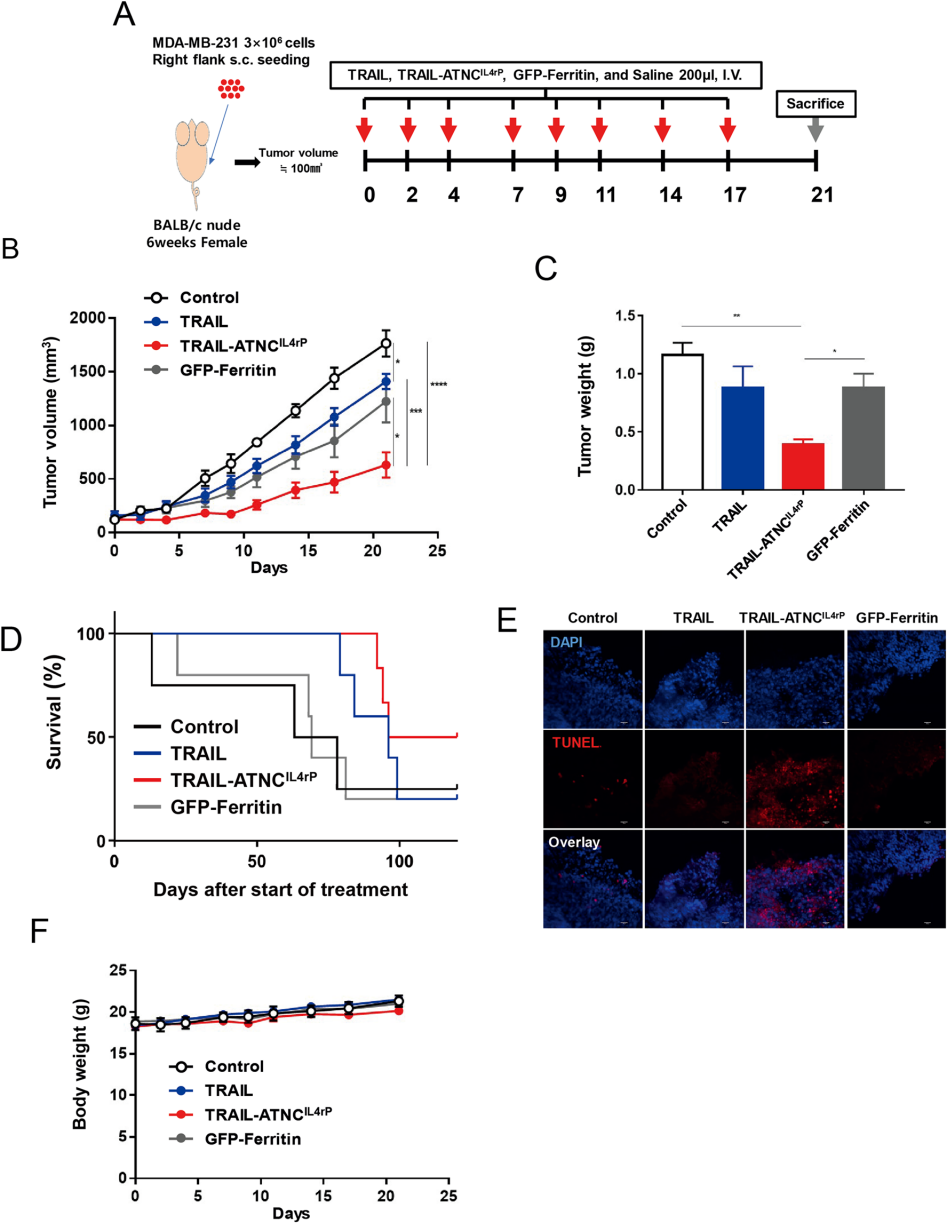
Anti-tumor effect of the TRAIL-ATNC^IL4rP^ in MDA-MB-231 tumor bearing mice. (**A**) Schematic diagram of MDA-MB-231 xenograft model experiment. MDA-MB-231 cells were injected subcutaneously to right flank of nude mice. After tumor volumes reached ~ 100 mm^3^, mice were treated with TRAIL-ATNC^IL4rP^ (10 mg/kg, n = 6), GFP-Ferritin (10 mg/kg, n = 7), TRAIL (5 mg/kg, equivalent to the number of moles of TRAIL in a dose of TRAIL-ATNC^IL4rP^, n = 7), or saline, pH 7.4 (control, n = 5) eight times every 2 or 3 d via intravenous injection. (**B**) Tumor growth rate in mice treated with the TRAIL-ATNC^IL4rP^, TRAIL, GFP-Ferritin, and saline. (**C**) Weights of excised tumors from each group at 21 d post-injection. Data represent means ± SEM (*p < 0.05, **p < 0.01, ***p < 0.001, and ****p < 0.0001; T-test) (**B**,**C**). (**D**) Kaplan–Meier survival curves after start of treatment. (**E**) Representative images of apoptotic cells, which were determined using TUNEL assays, in tumor sections from mice treated with TRAIL-ATNC^IL4rP^, TRAIL, GFP-Ferritin, or saline (control) (Scale bars: 30 µm). (**F**) The body weight change of each group during anti-tumor therapy.

We next evaluated the inhibition of tumor growth due to intravenous injection of TRAIL-ATNC^IL4rP^ compared with that of TRAIL and ferritin control. To accomplish this, we prepared a xenograft model by implanting MDA-MB-231 cells in mice, and then treated mice with TRAIL-ATNC^IL4rP^ (10 mg/kg), TRAIL (5 mg/kg, equivalent to the number of moles of TRAIL in a dose of TRAIL-ATNC^IL4rP^), or GFP-conjugated ferritin (GFP-Ferritin; 10 mg/kg, equivalent to the number of moles of ferritin in a dose of TRAIL-ATNC^IL4rP^) every 2 or 3 d after tumors had reached a volume of 100 mm^3^ (Fig. 6A). TRAIL-ATNC^IL4rP^ treated mice showed significantly inhibited tumor growth, whereas TRAIL and GFP-Ferritin treated groups showed minor tumor growth inhibition compared with saline controls (Fig. 6B,C). We also observed that compared to mice injected with TRAIL or GFP-Ferritin, mice injected with TRAIL-ATNC^IL4rP^ showed a significant decrease in tumor weight. Mice treated with TRAIL-ATNC^IL4rP^ every 2 days had its tumor volumes suppressed by 64.2%, while treatment of TRAIL had a much lower anti-cancer effect of 20.2%. Notably, this 3.2-fold greater efficacy was achieved by administering a molar dose of TRAIL-ATNC^IL4rP^ ~ 24-fold lower than that of TRAIL. The anti-tumor effect of TRAIL was even lower than that of GFP-Ferritin and this low efficacy of TRAIL in vivo is attributable to its instability and inefficient tumor targeting. Although GFP-ferritin treatment showed inhibition of tumor growth in vivo, there was a large variation between individual mice and no cytotoxic activity of GFP-ferritin was observed in vitro compared to TRAIL and TRAIL-ATNC^IL4rP^ (sFig. 13). GFP-ferritin can be passively accumulated in tumors by the EPR effect but the cause of anti-tumor activity of GFP-ferritin in vivo is not clear. Survival rate studies, however, showed that treatment with either TRAIL-ATNC^IL4rP^ or TRAIL resulted in higher survival rates than GFP-Ferritin or saline-treated control (Fig. 6D).

To confirm that the tumor growth inhibition induced by TRAIL-ATNC^IL4rP^ was driven by its apoptosis-promoting activity, we used terminal deoxynucleotidyl transferase dUTP nick-end labeling (TUNEL) staining to analyze apoptosis in tumor tissues from the mice that were treated as described above and euthanized on day 21 after the first injection. TRAIL-ATNC^IL4rP^ showed a greater percentage of TUNEL-positive cells compared to TRAIL, indicating a larger extent of apoptosis in tumor tissue (Fig. 6E). Quantification of TUNEL-positive tumor cells revealed a significantly higher percentage of apoptotic cells in the TRAIL-ATNC^IL4rP^ treatment group (73.2%) compared with the TRAIL treatment group (32.1%) (Fig. 6E). Taken together, these findings indicate that TRAIL-ATNC^IL4rP^, by virtue of its enhanced stability, higher affinity for the TRAIL receptor, and stronger apoptosis-promoting action, produces potent tumor cell apoptosis and thus successively inhibits tumor growth. No change in body weight and no liver or kidney damage were detected in all injected mice (Fig. 6F and sFig. 14A,B).

### In vivo pro-apoptotic activity of TRAIL-ATNC^IL4rP^ in an orthotopic pancreatic cancer model

**Figure 7:**
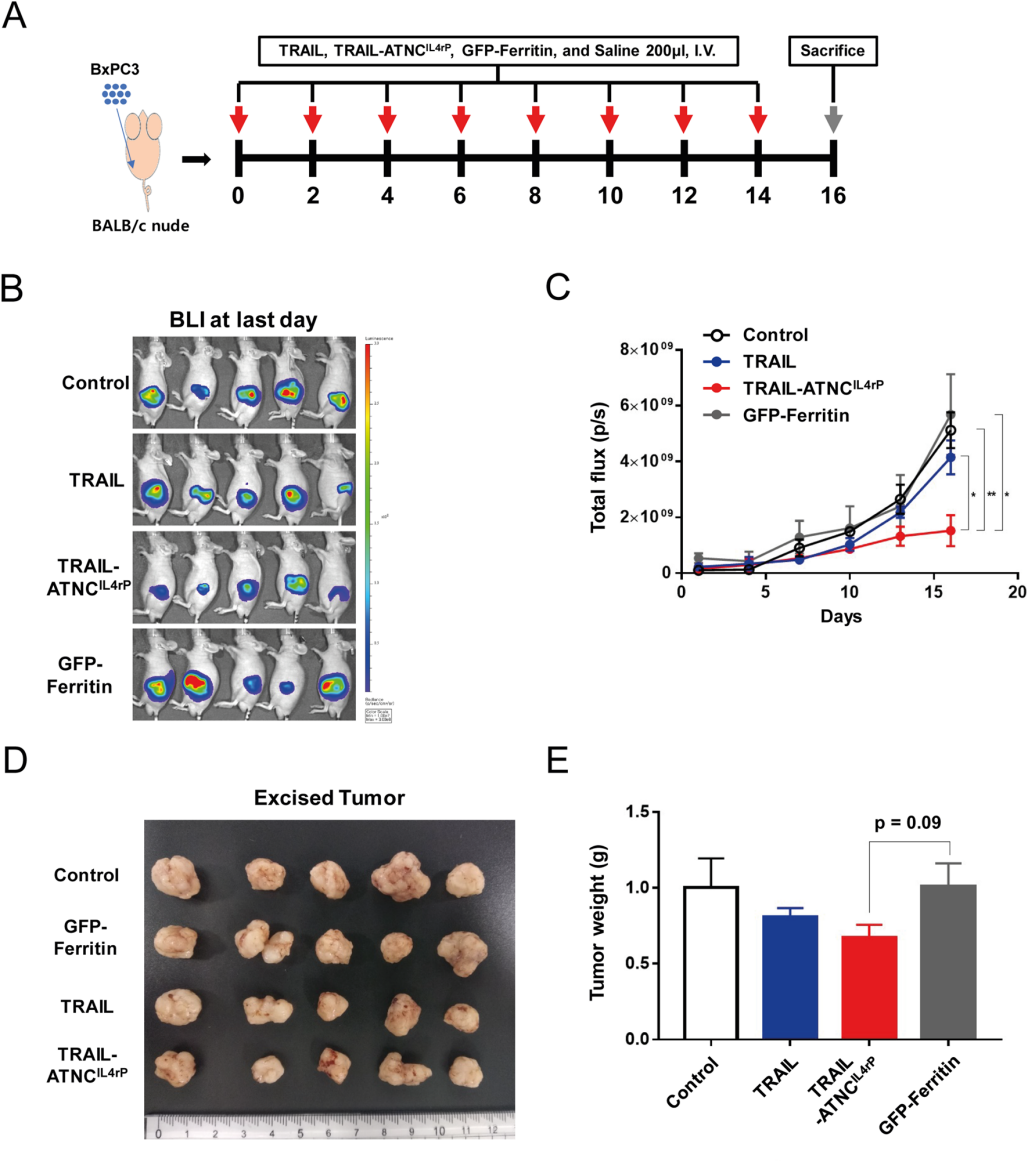
Anti-tumor effect of the TRAIL-ATNC^IL4rP^ in pancreatic tumor orthotopic mice model. (**A**) Schematic diagram of pancreatic tumor orthotopic model experiment. BxPC3 cells were surgically implanted into the pancreas of mice. After tumors grew, anti-tumor therapy was conducted by intravenously injecting Saline, pH7.4 (control, n = 5), TRAIL (5 mg/kg, n = 5), TRAIL-ATNC^IL4rP^ (8 mg/kg, n = 5), or GFP-Ferritin (5 mg/kg, n = 5) to tail vein every alternative days for a total of eight doses. (**B**) BLI at last day demonstrating lower luciferase readout in TRAIL-ATNC^IL4rP^ treated mice. (**C**) Tumor growth in pancreatic tumor orthotopic model treated with the TRAIL-ATNC^IL4rP^, TRAIL, GFP-Ferritin, or saline monitored using a bioluminescent IVIS imaging system. Data represent means ± SEM (*p < 0.05, **p < 0.01; T-test). (**D**) The images of the tumors collected from each mice group after treatment. (**E**) Weights of excised tumors from each group at 16 d post-injection. Data represent means ± SEM (p = 0.09; T-test).

Having confirmed the anti-tumor efficacy of TRAIL-ATNC^IL4rP^, we further verified whether this therapeutic strategy is applicable in another tumor model, namely pancreatic cancer, one of the most aggressive human neoplasms with an extremely poor prognosis and a low survival rate^66^. To test the anti-tumor efficacy of TRAIL-ATNC^IL4rP^, we first established orthotopic pancreatic tumor models by injecting luciferase-expressing BxPC3 cells, a TRAIL-sensitive human PDAC cell line^67^, into the pancreatic parenchyma of male BALB/c nude mice. Tumor-bearing mice were randomly divided into saline control, TRAIL, TRAIL-ATNC^IL4rP^ and GFP-Ferritin groups, then treated eight times with the corresponding formulations every 2 days beginning on the fourth day (Fig. 7A). Because tumor size could not be measured accurately in the orthotopic model, tumor growth was assessed by bioluminescence imaging (BLI) of tumors in each group (sFig. 15A and Fig. 7B,C).

On day 16, mice were sacrificed, and primary tumor volume and weight were measured together with an assessment of bioluminescence using an IVIS system (Fig. 7B,D,E and sFig. 15B). BLI analyses showed that treatment with TRAIL-ATNC^IL4rP^ inhibited primary tumor growth significantly more effectively than TRAIL compared with controls (saline or GFP-Ferritin). Tumors excised from TRAIL-ATNC^IL4rP^ treated mice trended smaller in terms of weight (p = 0.09) and volume (p = 0.12) compared with control mice. Although the size values were not statistically significant, the collective results with BLI suggest the potential therapeutic efficacy of TRAIL-ATNC^IL4rP^ against pancreatic tumors.

## Discussion

TRAIL has been considered a promising target for cancer therapy because it mediates activation of the extrinsic apoptosis pathway in a tumor-specific manner by binding to and trimerizing its functional receptors, DR4 or DR5. Many studies additionally indicated that the TRAIL receptors need to be clustered in order to transduce apoptotic signal efficiently^68^. After the initial interaction of trimeric TRAIL with three receptor molecules which are close together, a multimerization into supramolecular clusters occurred, which led to DISC assembly and caspase-8 activation. To date, both monomeric form of soluble TRAIL (114–281) and dimeric DR4/DR5 agonist monoclonal antibody (mAb) approaches have yielded suboptimal therapeutic outcomes, even in combination with conventional anti-cancer drugs. These failures are attributable to the limited ability of bivalent agonist mAbs to stimulate trimeric TRAIL receptors, which leads to inadequate DISC formation^69^, and the short half-life of the monomeric form of TRAIL, reflecting its intrinsic protein instability and rapid renal elimination^18^. A number of approaches have been tested for overcoming such obstacles encountered using native TRAIL. For example, a TRAIL-Trimer fusion protein, created using a trimer-tag from type I collagen, showed a half-life 3-times longer than that of native TRAIL in mice^62^. Recently, Kih et al*.* reported that a trimer-mimetic TRAIL on a nanocage exhibits superior binding affinity toward DR5 and anti-tumor efficacy compared with monomeric TRAIL, but no data around in vivo stability were not shown^70^.

We also utilized a trimer-tag—the triple helix from human pulmonary surfactant-associated protein D—and designed a TRAIL-Trimer fusion protein connected to the exposed threefold axis of the ferritin nanocage. The resulting TRAIL-ATNC exhibited potent apoptotic activity by virtue of its specific and high-affinity interactions (~ 235-fold higher than that of monomeric TRAIL) with DR4/DR5. This enhanced binding possibly comes from the increased avidity of multimeric TRAILs and the stabilization of a trimer form. TRAIL-ATNC showed dramatically improved cytotoxic activity compared to monomeric TRAIL or TRAIL-_GGGSG_-helix trimer, implying that multimeric display of TRAIL is the main cause for increased cytotoxic activity of TRAIL-ATNC. There is a report that the transmembrane form of TRAIL, but not soluble TRAIL, can induce supramolecular clusters and elicit suitable apoptotic signals, suggesting that spatial fixation and stabilization of the trimeric TRAIL structure is needed to transmit an apoptotic signal^71^. The multimeric TRAILs on the TRAIL-ATNC may have an advantage in supramolecular cluster formation of receptors to mediate apoptosis.

Although sub-populations of TRAIL-ferritin monomers seemed to fail to form 24-mer cages and some cages were aggregated in TRAIL-ATNC, pro-apoptotic activities of TRAIL-ATNC with or without IL4rP were 5- or 10- times stronger than that of TRAIL. Moreover, comparison of TRAIL-ATNCs with native TRAIL showed that the serum elimination half-life of TRAIL-ATNCs in mice was ~ 16-times longer than that observed for native TRAIL, suggesting improved in vivo stability of TRAIL-ATNC with or without IL4rP. In addition, the nano size of TRAIL-ATNC puts it above the limit for rapid elimination by renal filtration. A prolonged half-life of TRAIL-ATNC is a critical attribute because it results in greater systemic drug exposure at the tumor site. Furthermore, a number of previously developed TRAIL variants have been reported to exhibit dose limitations in clinical studies owing to instability in solution and rapid aggregation at high concentrations^17,18^.

The tumor-targeting efficiency of TRAIL-ATNC was significantly improved by multi-display of the tumor-targeting peptide, IL4rP, on the surface of the nanocage. This targeted TRAIL-ATNC^IL4rP^, which formed authentic cage structure compared to TRAIL-ATNC without IL4rP, conferred potent cytotoxic activity and in vivo stability similar to TRAL-ATNC without IL4rP. The TRAIL-ATNC^IL4rP^ showed dramatically enhanced tumor-targeting ability and superior anti-tumor efficacy in a xenograft breast cancer animal model. Recently, *Jyotsana *et al. reported that a low dose of leucocyte targeting TRAIL-liposome nanoparticles can kill the circulating cancer cells to decrease metastasis following tumor resection^72^. Therefore, TRAIL-ATNC can also be applied to cure metastatic cancer using a similar targeting strategy.

Most importantly, we tested the anti-tumor efficacy of the TRAIL-ATNC^IL4rP^ in an orthotopic pancreatic cancer model. PDAC remains one of the most aggressive cancers with a poor prognosis; thus, novel therapies are urgently needed. Most PDAC tumor cell lines are sensitive to TRAIL^73^, and preclinical studies suggest that PDACs are sensitive to TRAIL-mediated apoptosis in vitro and in vivo^74,75^. The native TRAIL formulation, dulanermin, was not clinically evaluated for PDAC, but agonistic TRAIL receptor Abs were shown to induce apoptosis in vitro and in pancreatic cancer xenograft models in vivo^76,77^. Phase II combination trials of TRAIL receptor Abs with gemcitabine, a conventional anti-cancer drug used to treat PDAC, have been performed in patients with unresectable or metastatic pancreatic cancer, and have shown similar therapeutic effect compared with gemcitabine monotherapy^78^. The superior cytotoxic efficacy of TRAIL-ATNC^IL4rP^ in vitro and in vivo and prolonged half-life in vivo suggest that TRAIL- ATNC^IL4rP^ may be able to overcome these drawbacks of native TRAIL or agonistic TRAIL receptor Abs. Indeed, in the current study, we found that TRAIL-ATNC^IL4rP^ exhibited marked anti-tumor activity in an orthotopic pancreatic cancer model. Although the concept of TRAIL as an important target against pancreatic cancer remains to be fully validated, our results underscore the promise of developing TRAIL active trimer nanocages as potential therapeutics in pancreatic cancer. Combination therapy with conventional anti-cancer drug could provide further assistance in overcoming TRAIL resistance in heterogeneous cancer cells.

Moreover, this active trimer delivery platform provides a scaffold for presenting other trimeric proteins such as TNF superfamily ligands or their receptors that share a trimeric assembly, extending the possible therapeutic applications of various ATNCs to different diseases.

## Methods

### Generation of TRAIL/IL4rP-conjugated ATNCs

The recombinant plasmids for the expression of the TRAIL Active Trimer NanoCage (ATNC) were constructed as described previously^79^. The TRAIL (114–281) gene was incorporated between NdeI and BamHI in pET-28a plasmid (69864-3, Novagen, MA, USA). The flexible linker (GGGSG) encoding oligonucleotide was synthesized and inserted between BamHI and EcoRI. The helix linker (EALQGQVQHLQAAFSQYKKVELFP), between EcoRI and SalI. The rigid linker (GGGGAEAAAKEAAAK), between SalI and SpeI. The short Ferritin heavy chain (sFtH, 15–161) gene was inserted between SpeI and HindIII. Interleukin 4 (IL4) receptor binding peptide (IL4rP, CRKRLDRNC) encoding oligonucleotide was synthesized and inserted after sFtH, between HindIII and XhoI. The Flexible linker and matrix metalloproteinase 2 cleavage site (GPLGLAG) and the flexible linker were synthesized and inserted between sFtH and IL4rP sequence.

### Protein expression and purification

The proteins were overexpressed in *E. coli* strain BL21 (DE3) cells and purified as described previously^55^. Briefly, the protein expression was induced by 0.1 mM isopropyl β-d-1-thiogalactopyranoside (IPTG) at 18 ℃ for 24 h when the cells were grown at 37 ℃ to an optical density at 600 nm (OD600) of 0.5. The expressed proteins were purified from the cell lysate using the Ni–NTA agarose bead (ThermoFisher Scientific, MA, USA) and was washed with the 0.1% Triton X-114 containing wash buffer (20 mM Tris pH 7.4, 500 mM NaCl, 30 mM imidazole, 0.5 mM DTT) to eliminate endotoxin^80^. After clearing the detergent by excess wash, the proteins were eluted by step-elution using buffers containing 100, 200, 300, and 500 mM imidazole in 20 mM Tris pH 7.4, 500 mM NaCl, and 1 mM DTT. The remained concentration of endotoxin in the purified protein fraction was calculated as ~ 9.3 EU/ml, indicating that ~ 98% of endotoxin was removed. The purified proteins were analyzed by sodium dodecyl sulfate–polyacrylamide gel electrophoresis (SDS-PAGE) to examine the homogeneity. To prepare fluorescence labeled proteins, TRAIL, TRAIL-ATNC, and TRAIL-ATNC^IL4rP^ were conjugated with NIR dye FPI-774 (λ_max_ ex/em, 782/814 nm) (Bioacts, Korea) according to the manufacturer’s protocol.

### Physicochemical of TRAIL-ATNCs

The purified TRAIL-ATNC and TRAIL-ATNC^IL4rP^ were analyzed using the dynamic light scattering (DLS) instrument (ELS-Z, Otzuka Electronics, Japan) and by transmission electron microscopy (TEM) as previously described^55^. For TEM analysis, protein samples were diluted to 0.25 mg/mL in buffer (20 mM Tris pH 7.4, 500 mM NaCl, 250 mM Imidazole, 1 mM DTT). Images were acquired using an FEI Tecnai at the Korea Institute of Science and Technology (KIST).

### Cell culture

Human breast adenocarcinoma MDA-MB-231 cells (ATCC, Manassas, VA) was cultured in high-glucose Dulbecco's modified Eagle's medium (DMEM) supplemented with 10% fetal bovine serum (FBS), 100 units/mL penicillin, and 100 μg/mL streptomycin. Human lung cancer cell A549 (ATCC, Manassas, VA) and H1703 (ATCC, Manassas, VA) were cultured in RPMI-1640 medium supplemented with 10% FBS, 100 units/mL penicillin, and 100 μg/mL streptomycin. Human PDAC cell line BxPC-3 (ATCC, Manassas, VA) were maintained in RPMI-1640 medium supplemented with 10% FBS, 100 units/mL penicillin, and 100 μg/mL streptomycin.

### In vitro apoptotic activity of TRAIL-ATNC

The MDA-MB-231 cells (5 × 10^3^ cells/well) were seeded on 96-well plates, grown in DMEM supplemented with 2% FBS for 24 h, and incubated with TRAIL, TRAIL-ATNC or TRAIL-ATNC^IL4rP^ (0–10 nM) for additional 24 h. The cell viability was evaluated using CellCountEZ Cell Survival Assay Kit (Rockland, USA) according to the manufacturer’s protocol. As a control of apoptosis, etoposide-treated (50 μM, Sigma) cells were monitored. The 50% inhibitory concentration (IC_50_) values were calculated by regression analysis using GraphPad Prism 7.0 Software (GraphPad Inc., USA). To validate apoptosis, the MDA-MB-231 cells (1 × 10^5^ cells/well) were incubated with TRAIL and TRAIL-ATNC (0–2.5 nM) at 37 °C for 24 h. After wash with PBS and binding buffer (10 mM HEPES pH 7.4, 140 mM NaCl, 2.5 mM CaCl_2_), the cells were incubated with annexin V-FITC and propidium iodide (PI) for 15 min at room temperature and subjected to FACS analysis (FACS Calibur cytometry, BD Biosciences) following the manufacturer’s protocol.

### Antibody blocking assay

The MDA-MB-231 cells (1 × 10^5^ cells) were seeded in 35-mm cell culture dishes and cultured for 24 h. For blocking TRAIL-mediated interaction, anti-DR4 antibody (AF347, R&D systems, Minneapolis, MN, USA; 200 ng/mL) or anti-DR5 antibody (CDM234, Cellsciences, Newburyport, MA, USA; 5 µg/mL) were pretreated to cells for 1 h in 37 ℃, and TRAIL (2.5 nM) or TRAIL-ATNC (0.3125 nM) were added and incubated for 3 h in 37 ℃. As a control, normal goat immunoglobulin G (Santa cruz, Dallas, TX, USA; 200 ng/mL) and normal mouse immunoglobulin G (Santa cruz, Dallas, TX, USA; 5 µg/mL) were used. To evaluate blocking effect of antibodies, the apoptotic cell percentage was measured as above.

### Cell binding analysis

Expression of TRAIL receptors on the surface of MDA-MB-231, A549, and H1703 cells were evaluated using FACS Calibur cytometry (BD Biosciences, San Jose, CA) with the following four anti-human TRAIL receptor antibodies: MAB347 (anti-DR4), MAB6311 (anti-DR5), MAB6302 (anti-DcR1), and MAB633(anti-DcR2) (R&D Systems, Minneapolis, MN, USA)^70^. For cell binding analyses, A549 cells (4 × 10^5^ cells) were seeded in eight chamber culture slides and grown for 24 h, followed by incubation with RPMI-1640 media containing 2% bovine serum albumin (BSA) at room temperature for 1 h. Cells were incubated with 1.2 μM TRAIL, 50 nM TRAIL-ATNC, or 50 nM Ferritin wild type at 4 ℃ for 1 h. It detected with Alexa Fluor 647 conjugated mouse anti-6 × His tag antibody (ThermoFisher Scientific, MA, USA). Cells were fixed with 4% paraformaldehyde (PFA), the nuclei were stained with DAPI, and analyzed under the confocal microscopy (K1-Fluo RT, Nanoscope Systems Inc., Daejeon, South Korea).

### Surface plasmon resonance analysis

Interactions of TRAIL or TRAIL-ATNC with TRAIL receptor, DR5, were analyzed using a surface plasmon resonance instrument (SR7500 DC, Reichert Inc., NY, USA) as described previously^43^. The extracellular region (1–182) of the DR5 protein (10465-H08H, Sino Biological, Beijing, China) was immobilized by activating the carboxymethyl group on dextran-coated chips through a reaction with a mixture of N-(3-dimethylaminopropyl)-N_0_-ethylcarbodiimide hydrochloride and N-hydroxysuccinimide (Sigma-Aldrich, St. Louis, MO, USA). Different concentrations of TRAIL-ATNC (0.52 to 8.33 nM) and TRAIL (25 to 400 nM) in binding buffer (100 mM Tris pH 7.4, 150 mM NaCl, 0.005% Tween20, 1 mM DTT) were allowed to flow over surfaces containing immobilized DR5 (585 RU) at a rate of 25 μl/min at 25 ℃. The sensor surface was regenerated after each association and dissociation cycle by injecting 2 M NaCl and 10 mM HCl for 1 min and 15 s, respectively. Sensorgrams were fit to a simple 1:1 Langmuir interaction model using data analysis program Scrubber 2.0 (BioLogic Software, Australia).

### Animal

All animal experiments with mice were performed in compliance with institutional guidelines and according to the animal protocol approved based on the guidelines of the Institutional Animal Care and Use Committee (IACUC) of Kyungpook National University (permission No. KNU 2018-0174) and the International Animal Care and Use Committee (IACUC) of the Laboratory of Animal Research at the Asan Medical Center, Seoul, Korea. (Permit Number: 2019-14-014). All efforts were made to minimize animal suffering. Female or male BALB/c nude mice (5 weeks old) and BALB/c wild type mice (8 weeks old) were purchased from Orient Bio Inc. (Seongnam, Rep of Korea). All mice bred in a pathogen free animal facility.

### Pharmacokinetic analysis of TRAIL and TRAIL-ATNC

Native TRAIL (0.01 mg/mL), TRAIL-ATNC (1 mg/mL), or TRAIL-ATNC^IL4rP^ (1 mg/mL) in saline (200 µL) were administrated using retro orbital injection into BALB/c wild type mice (n = 3 mice per group). Blood samples (25–30 μL) were collected at 5, 15, 30, 60, 180, 360, 720 min, and 24 h from each animal using heparinized tips. Plasma samples separated from mice blood, treated with TRAIL-ATNC, TRAIL-ATNC^IL4rP^, or native TRAIL, were diluted 1:1 in 5 × SDS-PAGE sample buffer, subjected to SDS-PAGE, analyzed by western blotting using anti-TRAIL antibody (ab9959, Abcam, USA), and quantified using Image J software. The serum from the untreated animals were used as control.

### In vivo tumor targeting and bio-distribution of TRAIL-ATNC^IL4rP^

The mice were subcutaneously injected in the flank with freshly harvested MDA-MB-231 cells (1 × 10^6^ cells/mouse) for the construction of the tumor xenograft model. FPI774-labeled TRAIL (0.54 mg/mL), TRAIL-ATNC (0.5 mg/mL), and TRAIL-ATNC^IL4rP^ (0.31 mg/mL), were intravenously injected into the MDA-MB-231 bearing mice (n = 3 mice/group) via the tail vein. Proteins containing equal amounts of fluorescence were injected so as to allow direct comparisons of tumor-targeting efficiency. Images were taken on IVIS Lumina imaging system (Caliper, USA) at 1, 6, 12 and 24 h post injection. To maintain sedation, animals received isofluorane continuously during the procedure. After 24 h, tumors and major organs were excised and analyzed using an IVIS Lumina imaging system (Caliper, USA). For analysis of fluorescence intensity in tumors, total photons per square centimeter per steradian (p/s/cm^2^/sr) in the region of interest (ROI) were measured and calculated using an IVIS Lumina imaging system (Caliper, USA) and Living Image Software.

### In vivo anti-tumor efficacy in breast cancer xenograft model

An in vivo tumor model was established by subcutaneously injecting MDA-MB-231 cells (3 × 10^6^ cells/mouse) into the dorsal flank of female BALB/c nude mice and allowing tumors to grow for 2 weeks (~ 100 mm^3^). Tumor-bearing mice were subjected to randomization and divided into the four groups: TRAIL-ATNC^IL4rP^ (10 mg/kg, n = 6), GFP-Ferritin (10 mg/kg, n = 7), TRAIL (5 mg/kg, equivalent to the number of moles of TRAIL in a dose of TRAIL-ATNC^IL4rP^, n = 7), and saline (pH 7.4, n = 5). All treatments were administered by intravenous injection via the tail vein every 2 or 3 d for a total of eight doses. Tumor size was measured once every 2 or 3 d during the experimental period, and tumor volume was calculated using the following formula: Volume = (Length × Width × Width)/2. Percentage of survival was measured up to 120 days after the initiation of treatment. At the end of the treatment, tumors were dissected and weighed, then analyzed apoptosis by terminal deoxynucleotidyl transferase dUTP nick-end labeling (TUNEL) assay using the ApopTag Red In Situ Apoptosis Detection Kit in accordance with the manufacturer’s instructions (Millipore, Temecula, USA).

### In vivo anti-tumor efficacy in pancreatic cancer orthotropic model

The surgical procedures were performed in a specific-pathogen-free room. Six-week-old male BALB/c nude mice (n = 5 per group) were anaesthetized by intraperitoneal injection of 250 mg/kg 2, 2, 2-tribromoethanol (Sigma-Aldrich). The luciferase-transfected BxPC3 cells were surgically implanted into the pancreas of mice as described previously^81,82^. Briefly, freshly harvested BxPC3 cells (2 × 10^6^ cells in matrigel) were slowly injected into the pancreas using a 29 gauge needle. After slow removal of the needle, the injection site was held by a sterile cotton swab for 30 s to prevent leakage of the cell suspension into the abdomen. The pancreas and spleen were then returned to the appropriate position, and the skin and peritoneum were closed in one layer.

Pancreatic tumor development and growth were evaluated using a bioluminescent IVIS in vivo imaging System (IVIS Spectrum system, PerkinElmer, Hopkinton, MA). Imaging was taken 12 min after the injection of d-luciferin (150 mg/kg)^83^. The light emitted from luciferase expressing tumor cells was digitized and displayed as a pseudo-color image onto a gray scale animal image. When BLI suggested established tumor growth to start anti-tumor therapy, mice were randomly divided into 4 treatment groups. The tumor bearing mice were intravenously administrated with Saline (pH 7.4), TRAIL (5 mg/kg), TRAIL-ATNC^IL4rP^ (8 mg/kg), and GFP-Ferritin (5 mg/kg) on alternative days. Mice were followed for BLI readout at 1, 4, 7, 10, 13, 16 days. The mice were sacrificed on day 16, and the size of the tumors was calculated.

### Statistical analysis

Statistical significance was determined using a Student's t-test. Results are shown as mean ± SEM of at least three different experiments. P values ≤ 0.05 were considered statistically significant.

## Supplementary information

Supplementary Information.
