# Supplementary material for: Designed ferritin nanocages displaying trimeric TRAIL and tumor-targeting peptides confer superior anti-tumor efficacy
Source: Sci Rep. 2020 Nov 17;10:19997. doi: 10.1038/s41598-020-77095-x (PMC7672110; doi:10.1038/s41598-020-77095-x)
Supplement: Supplementary file 1 — Supplementary Information. [file 41598_2020_77095_MOESM1_ESM.pdf]

Supplementary materials

Designed ferritin nanocage displaying trimeric TRAIL and tumor targeting peptide confers superior anti-tumor efficacy.

Jae Do Yoo[a], Sang Mun Bae[b], Junyoung Seo[a], In Seon Jeon[a], Sri Murugan Poongkavithai Vadevoo[a], Sang-Yeob Kim[c,d], In-San Kim[e,f], Byungheon Lee[a], and Soyoun Kim[a*]

[a]Department of Biochemistry and Cell Biology, Cell and Matrix Research Institute, School of Medicine, Kyungpook National University, Daegu 41944, Republic of Korea.

[b]PrismCDX, Inc, 593-16, Dongtangiheung-ro, Hwaseong-si, Gyeonggi-do, 18469, Republic of Korea.

[c]Asan Institute for Life Sciences, Asan Medical Center, University of Ulsan College of Medicine, Seoul 138-736, South Korea

[d]Department of Convergence Medicine, University of Ulsan College of Medicine, Seoul 05505, South Korea.

[e]Biomedical Research Institute, Korea Institute of Science and Technology, Seoul 02792, Republic of Korea

[f]KU-KIST Graduate School of Converging Science and Technology, Korea University, Seoul 02841, Republic of Korea.

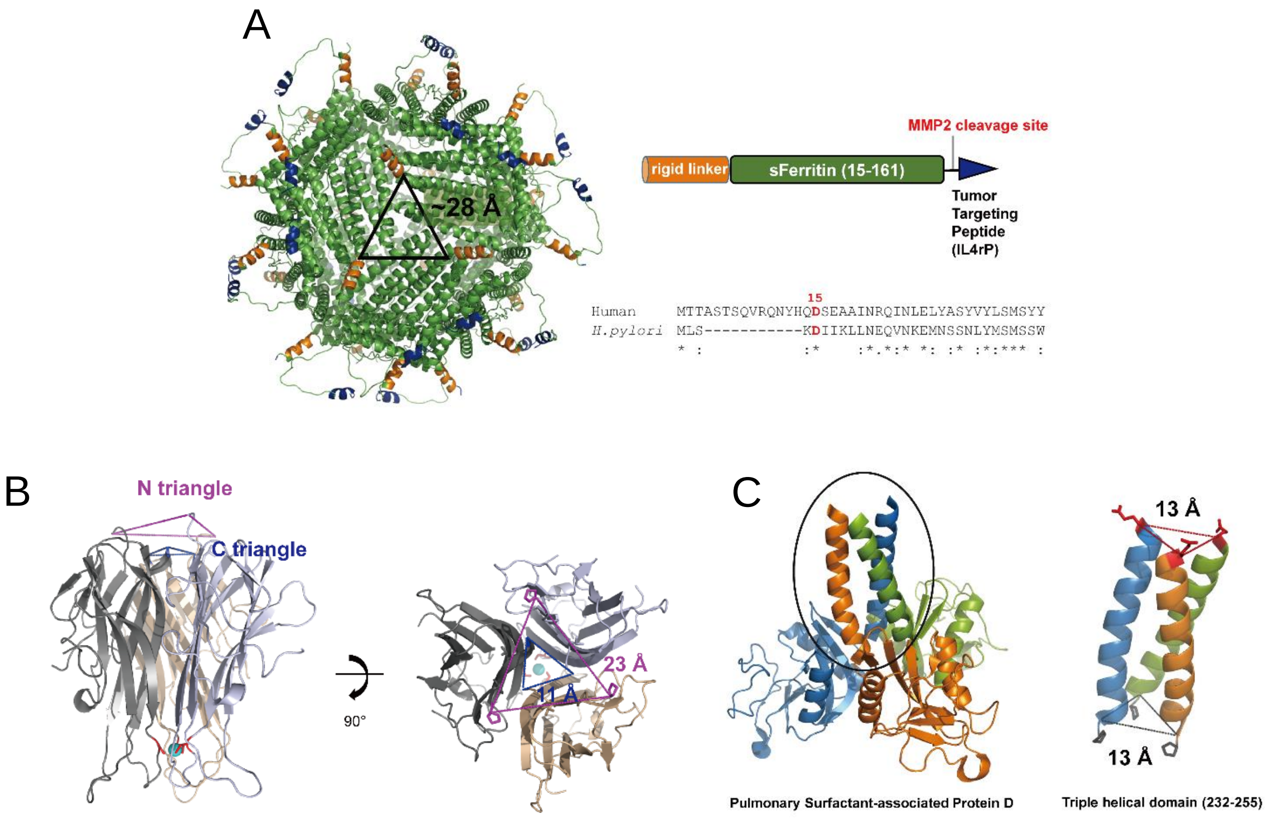

Figure S1. Design of the Active Trimer Nanocage (ATNC) platform

(A) Sequence alignment of human ferritin heavy chain with *H. pylori* ferritin indicates Asp15 of human is aligned to Asp5 of *H. pylori*. 3D structure model was built based on the crystal structure of human ferritin heavy chain (PDB 2FHA) showing the rigid helix linkers (orange) are fused at the trimeric position with a distance of 28 Å. (B) 3D protein structure of the ecto-domain of the TRAIL trimeric complex (PDB 1DG6) presents triangle position of N and C termini with a distance of 23 and 11 Å, respectively. (C) Triple helical domain from the pulmonary surfactant associated protein D (PDB 5OXS). The images (A-C) were drawn using PyMOL v0.99 (The PyMOL Molecular Graphics System, Schrödinger, LLC).

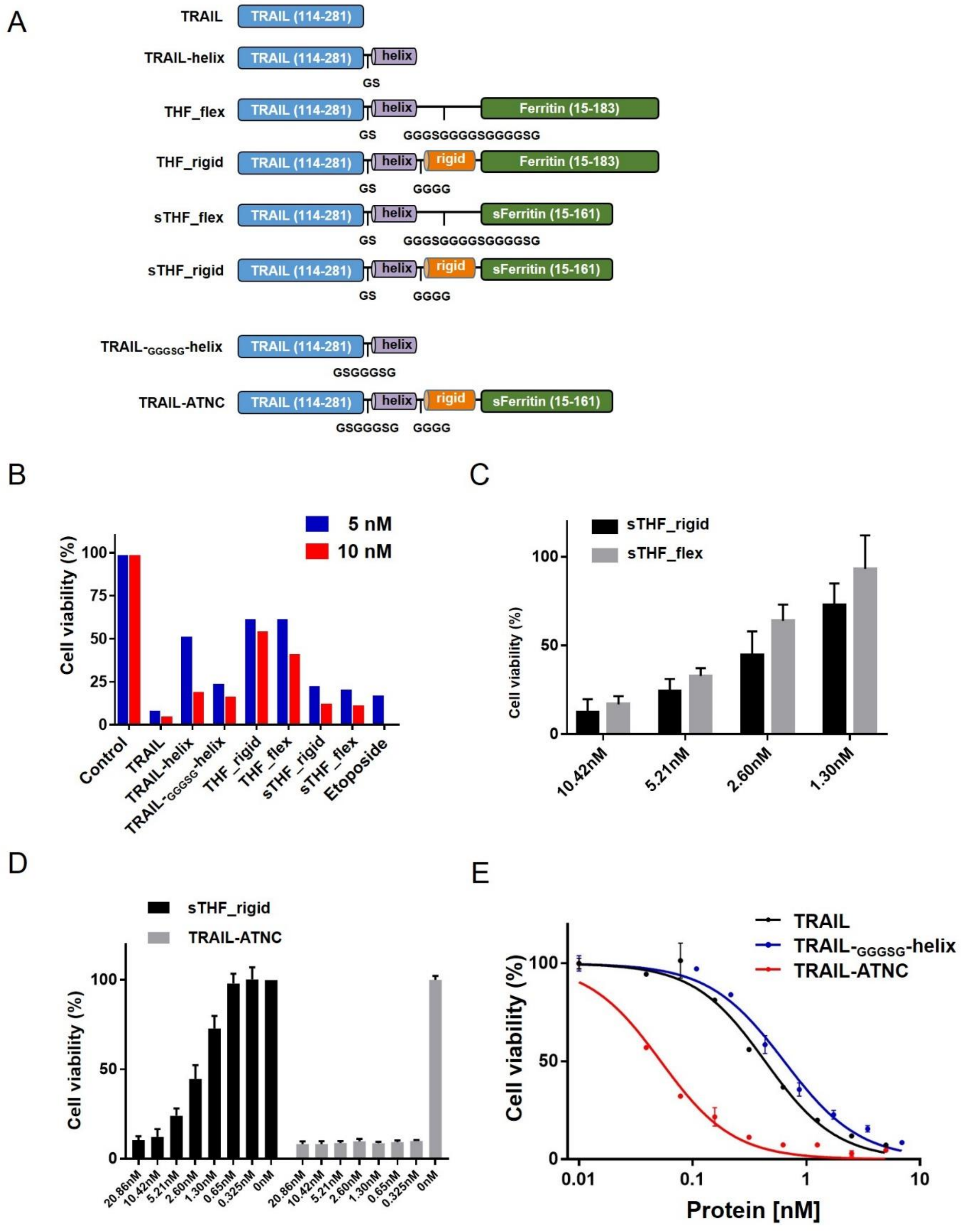

Figure S2. Cancer cell cytotoxicity of the TRAIL-ferritin fusion protein constructs.

(A) Schematic diagram of the TRAIL-ferritin fusion protein constructs. The helix linker is the triple helical domain from the pulmonary surfactant associated protein D. The rigid linker is

(EAAAK) × 2. The Flexible linker, (GGGSG) × 3, was showed. The final form of the TRAIL-ferritin fusion constructs was named as TRAIL-ATNC as shown. (B) MDA-MB-231 cancer cells were treated by various TRAIL-ferritin fusion proteins and the cytotoxicity was measured by EZcount MTT assay. Etoposide (50 uM) was treated as an apoptosis control. (C) MDA-MB-231 cells were treated by sTHF rigid *vs*. flex proteins. (D) MDA-MB-231 cells were treated by TRAIL-ATNC *vs*. sTHF rigid proteins. TRAIL-ATNC showed the most potent cytotoxicity. (E) Cell viability following treatment with TRAIL, TRAIL-$_{\text{GGGSG}}$-helix, or TRAIL-ATNC. MDA-MB-231 cells were incubated with proteins for 24 h and then measured by EZ-count assay. Data represent means ± SEM.

TRAIL-ATNC$^{\mathrm{IL4rP}}$

~6His~TRAIL(114-281)~Helix~rigid_linker~Ferritin(15-161)~MMP2~IL4rP

ATGGGCAGCAGC**CATCATCATCATCATCAC**AGCAGCGGCCTGGTGCCGCGCGGCAGCCAACATATGGTGAGAGAAAGAGGTCCTCAGAGA

M G S S **H H H H H H** S S G L V P R G S Q H M V R E R G P Q R

GTAGCAGCTCACATAACTGGGACCAGAGGAAGAAGCAACACATTGTCTTCTCCAAACTCCAAGAATGAAAAGGCTCTGGGCCGCAAAATA

V A A H I T G T R G R S N T L S S P N S K N E K A L G R K I

AACTCCTGGGAATCATCAAGGAGTGGGCATTCATTCCTGAGCAACTTGCACTTGAGGAATGGTGAACTGGTCATCCATGAAAAAGGGTTT

N S W E S S R S G H S F L S N L H L R N G E L V I H E K G F

TACTACATCTATTCCCAAACATACTTTCGATTTCAGGAGGAAATAAAAGAAAACACAAAGAACGACAAACAAATGGTCCAATATATTTAC

Y Y I Y S Q T Y F R F Q E E I K E N T K N D K Q M V Q Y I Y

AAATACACAAGTTATCCTGACCCTATATTGTTGATGAAAAGTGCTAGAAATAGTTGTTGGTCTAAAGATGCAGAATATGGACTCTATTCC

K Y T S Y P D P I L L M K S A R N S C W S K D A E Y G L Y S

ATCTATCAAGGGGGAATATTTGAGCTTAAGGAAAATGACAGAATTTTTGTTTCTGTAACAAATGAGCACTTGATAGACATGGACCATGAA

I Y Q G G I F E L K E N D R I F V S V T N E H L I D M D H E

GCCAGTTTTTTTGGGGCCTTTTTAGTTGGCGGATCCGGTGGAGGATCTGGTGAATTCGAAGCGTTACAGGGCCAGGTTCAGCACCTGCAG

A S F F G A F L V G G S G G G S G E F E A L Q G Q V Q H L Q

GCAGCTTTCAGCCAGTATAAAAAGGTAGAACTGTTTCCAGTCGACGGTGGAGGAGGTGCGGAGGCGGCGGCGAAGGAGGCGGCGGCGAAG

A A F S Q Y K K V E L F P V D G G G G A E A A A K E A A A K

ACTAGTGACTCAGAGGCCGCCATCAACCGCCAGATCAACCTGGAGCTCTACGCCTCCTACGTTTACCTGTCCATGTCTTACTACTTTGAC

T S D S E A A I N R Q I N L E L Y A S Y V Y L S M S Y Y F D

CGCGATGATGTGGCTTTGAAGAACTTTGCCAAATACTTTCTTCACCAATCTCATGAGGAGAGGGAACATGCTGAGAAACTGATGAAGCTG

R D D V A L K N F A K Y F L H Q S H E E R E H A E K L M K L

CAGAACCAACGAGGTGGCCGAATCTTCCTTCAGGATATCAAGAAACCAGACTGTGATGACTGGGAGAGCGGGCTGAATGCAATGGAGTGT

Q N Q R G G R I F L Q D I K K P D C D D W E S G L N A M E C

GCATTACATTTGGAAAAAAATGTGAATCAGTCACTACTGGAACTGCACAAACTGGCCACTGACAAAAATGACCCCCATTTGTGTGACTTC

A L H L E K N V N Q S L L E L H K L A T D K N D P H L C D F

ATTGAGACACATTACCTGAATGAGCAGGTGAAAGCCATCAAAGAATTGGGTGACCACGTGACCAACTTGCGCAAGATGGGAGCGAAGCTT

I E T H Y L N E Q V K A I K E L G D H V T N L R K M G A K L

GGTGGAGGATCTGGTGGACCACTGGGACTGGCGGGCGGTGGAGGATCTGGTTGCCGTAAACGTCTGGATCGTAACTGC**TAA**

G G G S G G P L G L A G G G G S G C R K R L D R N C *

Figure S3. Sequence of TRAIL-ATNC$^{\mathrm{IL4rP}}$

TRAIL-ATNC

~6His~TRAIL(114-281)~Helix~rigid_linker~Ferritin(15-161)

ATGGGCAGCAGCCATCATCATCATCATCACAGCAGCGGCCTGGTGCCGCGCGGCAGCCAACATATGGTGAGAGAAAGAGGTCCTCAGAGA
M G S S H H H H H H S S G L V P R G S Q H M V R E R G P Q R

GTAGCAGCTCACATAACTGGGACCAGAGGAAGAAGCAACACATTGTCTTCTCCAAACTCCAAGAATGAAAAGGCTCTGGGCCGCAAAATA
V A A H I T G T R G R S N T L S S P N S K N E K A L G R K I

AACTCCTGGGAATCATCAAGGAGTGGGCATTCATTCCTGAGCAACTTGCACTTGAGGAATGGTGAACTGGTCATCCATGAAAAAGGGTTT
N S W E S S R S G H S F L S N L H L R N G E L V I H E K G F

TACTACATCTATTCCCAAACATACTTTCGATTTCAGGAGGAAATAAAAGAAAACACAAAGAACGACAAACAAATGGTCCAATATATTTAC
Y Y I Y S Q T Y F R F Q E E I K E N T K N D K Q M V Q Y I Y

AAATACACAAGTTATCCTGACCCTATATTGTTGATGAAAAGTGCTAGAAATAGTTGTTGGTCTAAAGATGCAGAATATGGACTCTATTCC
K Y T S Y P D P I L L M K S A R N S C W S K D A E Y G L Y S

ATCTATCAAGGGGGAATATTTGAGCTTAAGGAAAATGACAGAATTTTTGTTTCTGTAACAAATGAGCACTTGATAGACATGGACCATGAA
I Y Q G G I F E L K E N D R I F V S V T N E H L I D M D H E

GCCAGTTTTTTTGGGGCCTTTTTAGTTGGCGGATCCGGTGGAGGATCTGGTGAATTCGAAGCGTTACAGGGCCAGGTTCAGCACCTGCAG
A S F F G A F L V G G S G G G S G E F E A L Q G Q V Q H L Q

GCAGCTTTCAGCCAGTATAAAAAGGTAGAACTGTTTCCAGTCGACGGTGGAGGAGGTGCGGAGGCGGCGGCGAAGGAGGCGGCGGCGAAG
A A F S Q Y K K V E L F P V D G G G G A E A A A K E A A A K

ACTAGTGACTCAGAGGCCGCCATCAACCGCCAGATCAACCTGGAGCTCTACGCCTCCTACGTTTACCTGTCCATGTCTTACTACTTTGAC
T S D S E A A I N R Q I N L E L Y A S Y V Y L S M S Y Y F D

CGCGATGATGTGGCTTTGAAGAACTTTGCCAAATACTTTCTTCACCAATCTCATGAGGAGAGGGAACATGCTGAGAAACTGATGAAGCTG
R D D V A L K N F A K Y F L H Q S H E E R E H A E K L M K L

CAGAACCAACGAGGTGGCCGAATCTTCCTTCAGGATATCAAGAAACCAGACTGTGATGACTGGGAGAGCGGGCTGAATGCAATGGAGTGT
Q N Q R G G R I F L Q D I K K P D C D D W E S G L N A M E C

GCATTACATTTGGAAAAAAATGTGAATCAGTCACTACTGGAACTGCACAAACTGGCCACTGACAAAAATGACCCCCATTTGTGTGACTTC
A L H L E K N V N Q S L L E L H K L A T D K N D P H L C D F

ATTGAGACACATTACCTGAATGAGCAGGTGAAAGCCATCAAAGAATTGGGTGACCACGTGACCAACTTGCGCAAGATGGGAGCGTAA
I E T H Y L N E Q V K A I K E L G D H V T N L R K M G A *

Figure S4. Sequence of TRAIL-ATNC

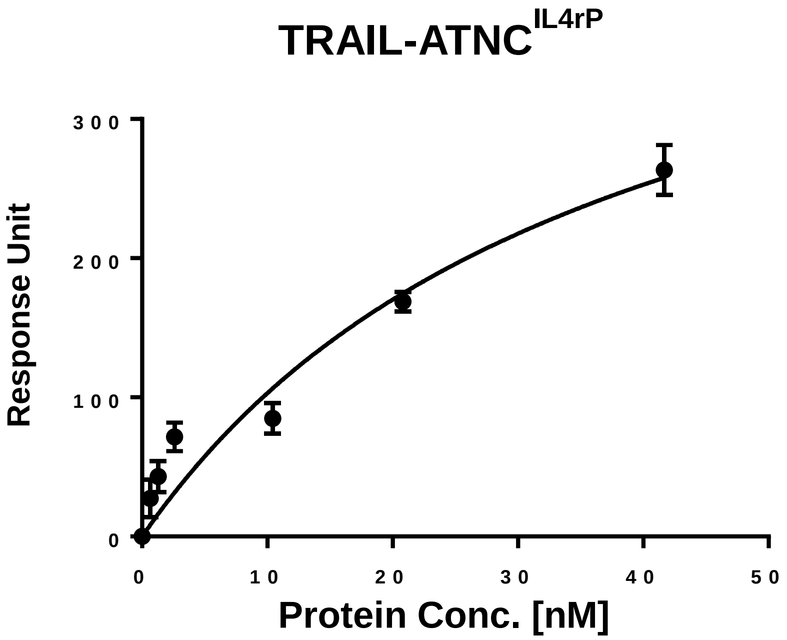

Figure S5. Binding kinetics of TRAIL-ATNC^{IL4rP} with IL-4 receptor.

Surface plasmon resonance analysis for the binding kinetics of TRAIL-ATNC^{IL4rP} with IL-4 receptor. The concentrations of TRAIL-ATNC^{IL4rP} (1.25-40 nM) were injected to IL-4 receptor α domain coated dextran-coated chips. The IL-4 receptor α domain (1-232 amino acid) was purchased (10402-H08H, Sino Biological, Beijing, China). The steady-state binding data were analyzed by fitting the curve of binding level against concentration with a 1:1 binding model (GraphPad Prism 7.0 software). K_D was estimated as 37.67 ± 3.348 nM. Data were collected from 3 independent experiments (error bar represents S.D).

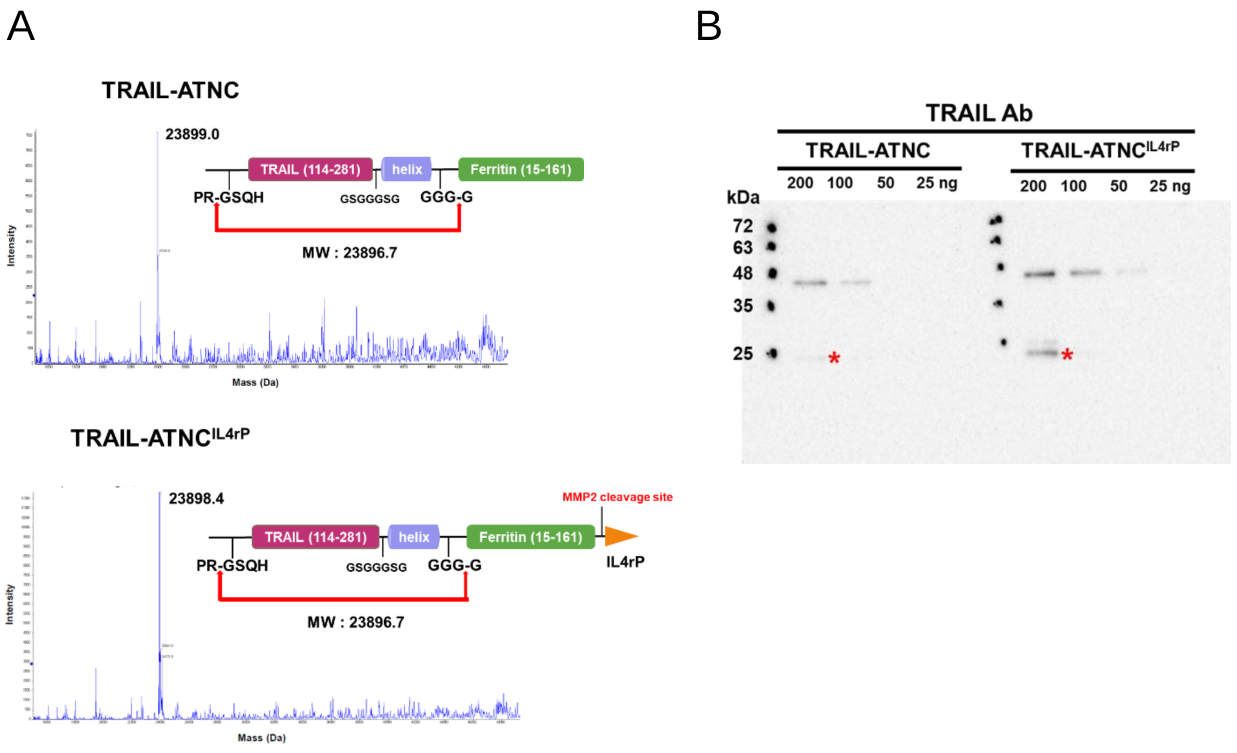

Figure S6. Mass spectroscopy analysis of TRAIL-ATNC and TRAIL-ATNC^{IL4rP}

(A) The purified TRAIL-ATNC and TRAIL-ATNC^{IL4rP} were analyzed by mass spectroscopy (LC-ESI Q-TOF). A peak (MW = 23899.0) of the TRAIL-ATNC and one (MW = 23898.4) of TRAIL-ATNC^{IL4rP} were detected. Schematic diagrams showed the cleavage sites (thrombin cleavage site (LVPR^{18}GS) on N-terminal vector sequence of TRAIL and the flexible linker region (GG^{228}GG) between helix and ferritin) of the best matched fragments (amino acids 18-228) from TRAIL-ATNC constructs. (B) The western blot analysis with anti-TRAIL Ab showed TRAIL-containing fragments (red *) from TRAIL-ATNC and TRAIL-ATNC^{IL4rP}.

A

B

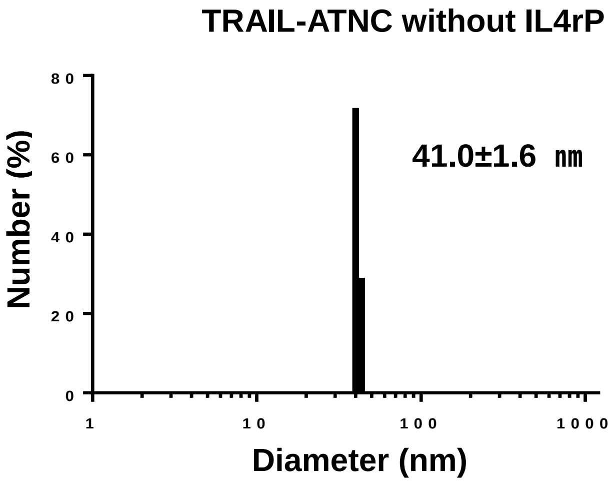

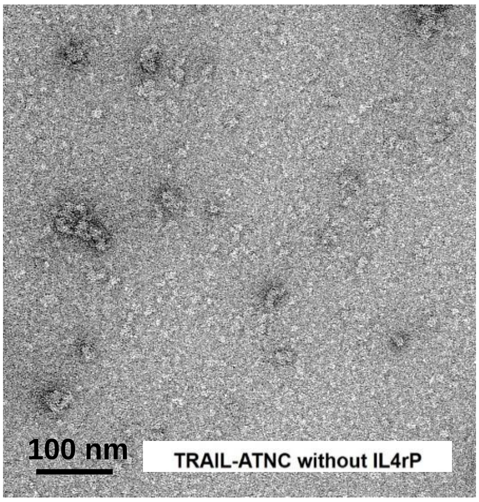

Figure S7. Physicochemical characterization of TRAIL-ATNC without IL4rP

(A) DLS analysis of TRAIL-ATNC without IL4rP showing homogeneous, self-assembled, nano-sized particles. (B) Transmission electron microscopy image of uracil acetate stained TRAIL-ATNC without IL4rP.

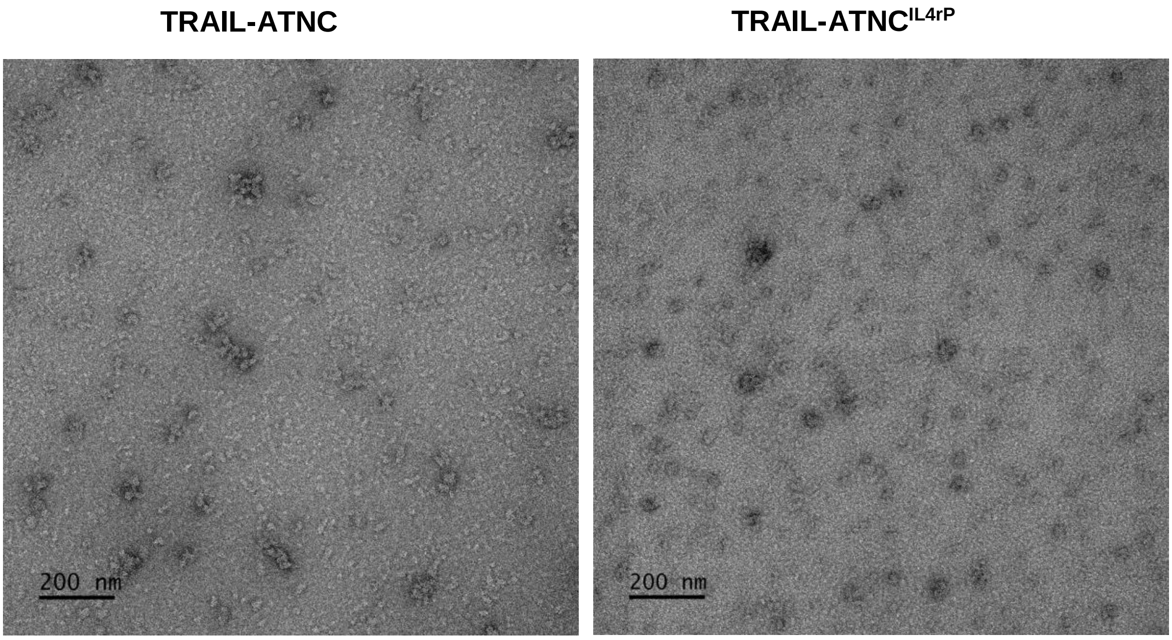

Figure S8. Low magnification TEM images of TRAIL-ATNC and TRAIL-ATNC^{IL4rP}

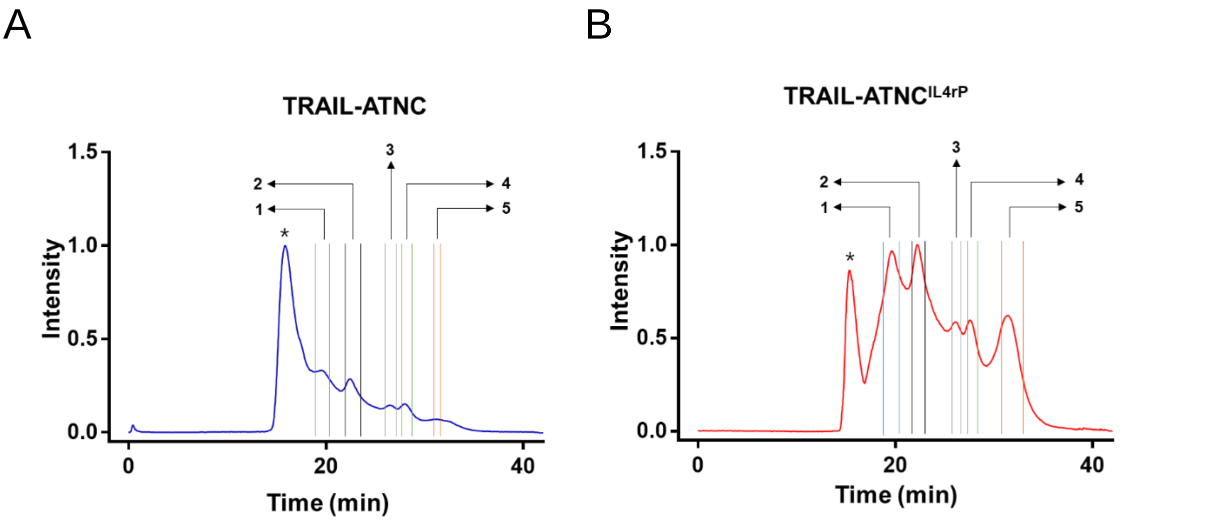

Peak	*	1	2	3	4	5
Time (min)	15.8	19.5	22.5	26.4	27.9	31.3
MW(kDa)	n/a	798.8	307.9	124.8	91.03	54.03

Peak	*	1	2	3	4	5
Time (min)	15.3	19.6	22.2	26.1	27.5	31.4
MW(kDa)	n/a	640.2	284.3	118.1	85.76	42.89

Figure S9. Multiangle Light Scattering (MLS) analysis of TRAIL-ATNC and TRAIL-ATNC^{IL4rP}.

The particle formation and heterogeneity were analyzed using the Multiangle Light Scattering (MLS) for TRAIL-ATNC (A) and TRAIL-ATNC^{IL4rP} (B). Both TRAIL-ATNC and TRAIL-ATNC^{IL4rP} formed 24-mer cage (no. 1), estimated as 798.8 and 640.2 kDa, respectively. Multiple smaller sized peaks were observed which are likely monomer (no. 5), dimer (no. 4), trimer (no. 3), and hexamer (no. 2) conformation. *aggregated particles.

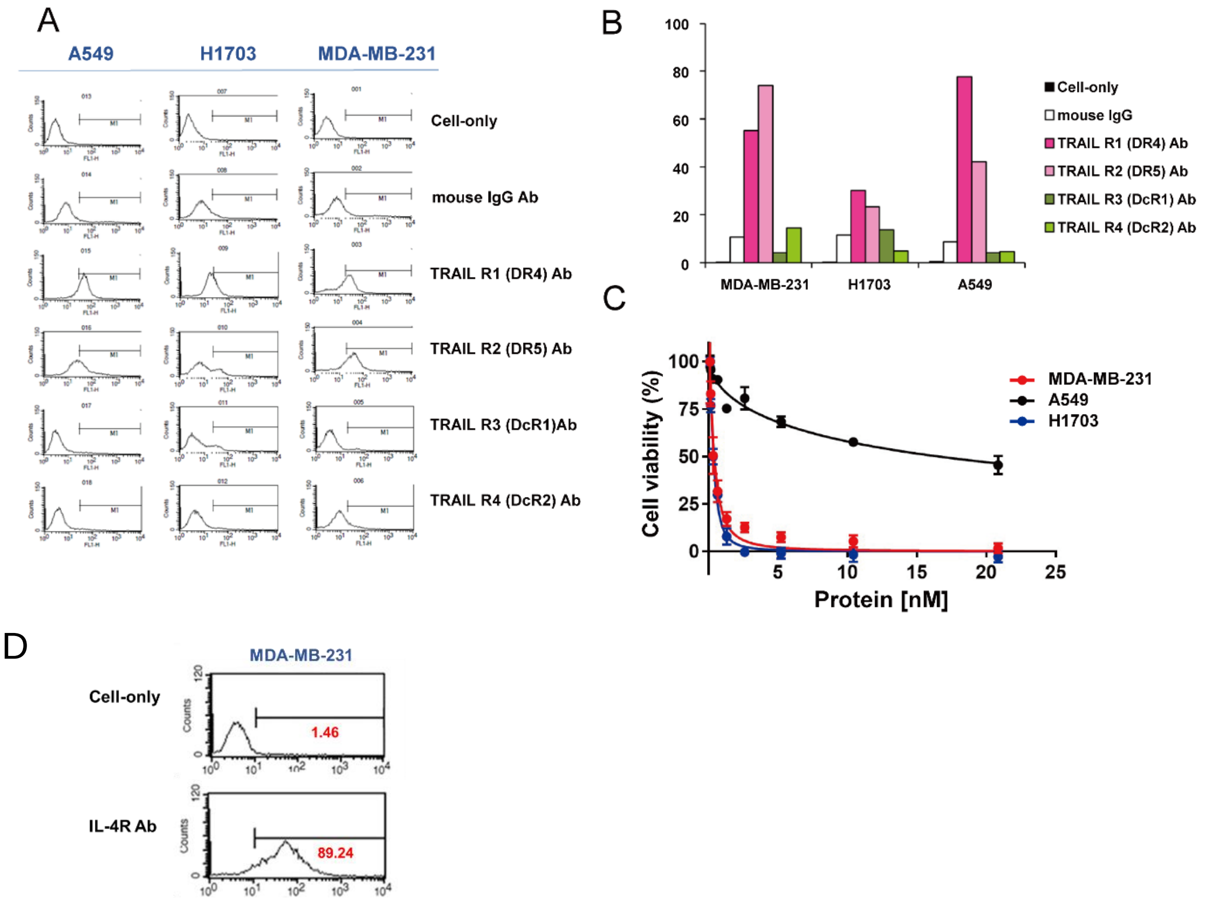

Figure S10. TRAIL receptor expression and TRAIL sensitivity in various cancer cell lines.

(A and B) The expression levels of TRAIL receptors were evaluated in MDA-MB-231, A549, and H1703 cell line using anti-human TRAIL receptor antibodies, MAB347 (anti-DR4), MAB6311 (anti-DR5), MAB6302 (anti-DcR1), and MAB633 (anti-DcR2) (R&D Systems), by flow cytometry. (C) The TRAIL sensitivity was monitored in MDA-MB-231, A549, and H1703 cells by in vitro cytotoxicity assay. (D) The expression levels of IL4 receptors were evaluated in MDA-MB-231 cell line using anti-human IL4 receptor antibodies (sc-28361, Santa cruz) by flow cytometry.

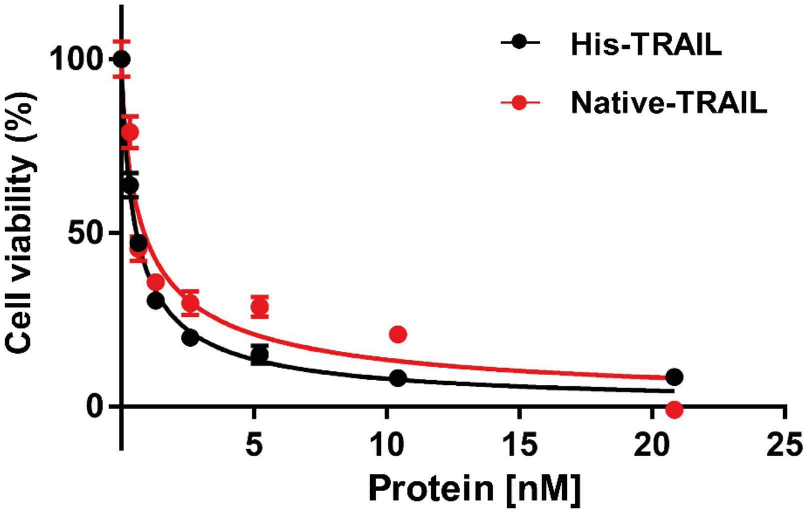

	IC_{50}
His-TRAIL	0.57 ± 0.04 nM
Native-TRAIL	0.86 ± 0.13 nM

Figure S11. Comparing cytotoxicity between His-TRAIL and Native-TRAIL

Cell cytotoxicity was compared between the His-tagged TRAIL and native TRAIL. MDA-MB-231 cells were incubated with concentrations of His-tagged TRAIL or Native TRAIL for 24 h and then measured by EZ-count assay. Estimated IC_{50} of His-tagged TRAIL and Native TRAIL were calculated using GraphPad Prism v7.0. Data represent means ± SEM.

A. TRAIL-ATNC B. Wildtype FtH

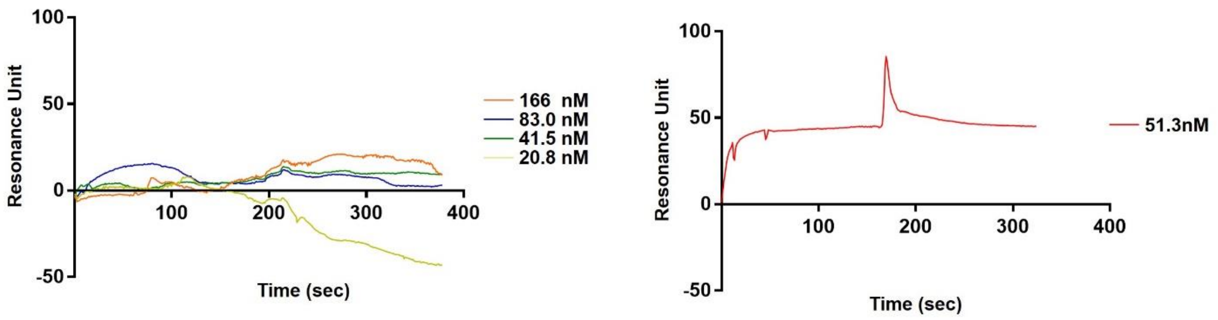

Figure S12. Surface plasmon resonance analysis for the binding of TRAIL-ATNC (A) or wildtype ferritin heavy chain (FtH) (B) with Transferrin receptor 1 (TfR1) receptor. The concentrations of TRAIL-ATNC (20.8~166 nM) and FtH (51.3 nM) were injected to TfR1 coated dextran-coated chips. The TfR1 ectodomain (89-760 amino acid) was purchased (12020-H08H, Sino Biological, Beijing, China).

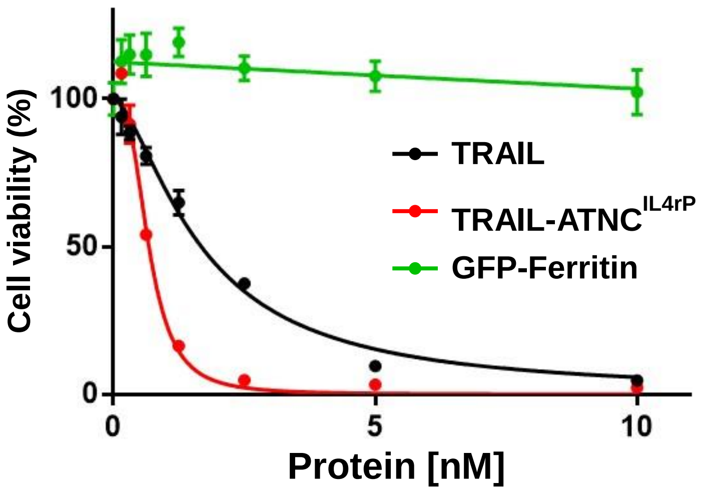

Figure S13. Comparing cytotoxicity among GFP-Ferritin, TRAIL, and TRAIL-ATNC^{IL4rP}

MDA-MB-231 cells were incubated with concentrations of GFP-Ferritin, TRAIL, and TRAIL-ATNC^{IL4rP} and then measured by EZ-count assay. Data represent means ± SD.

A

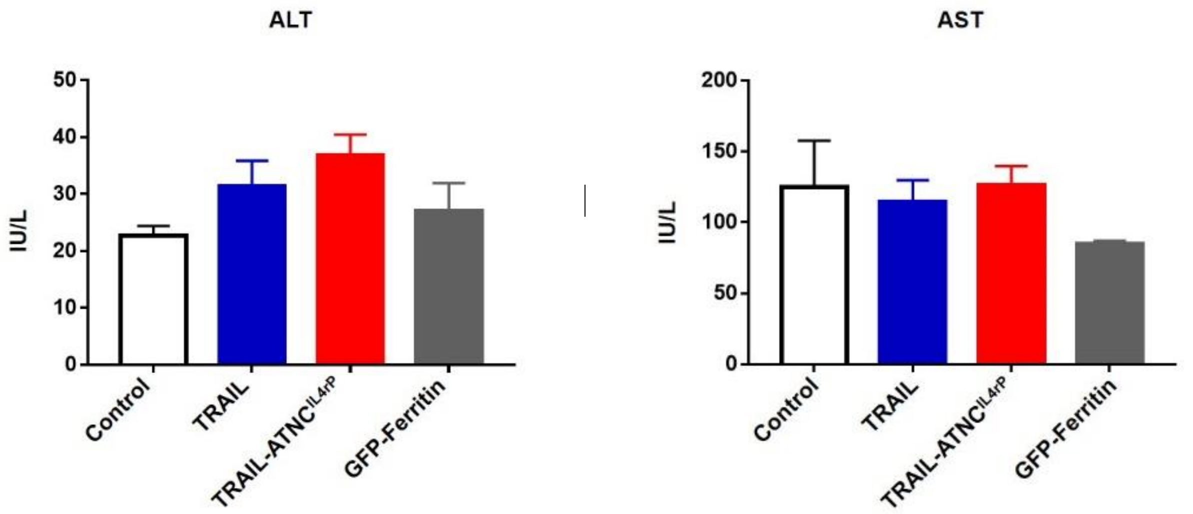

B

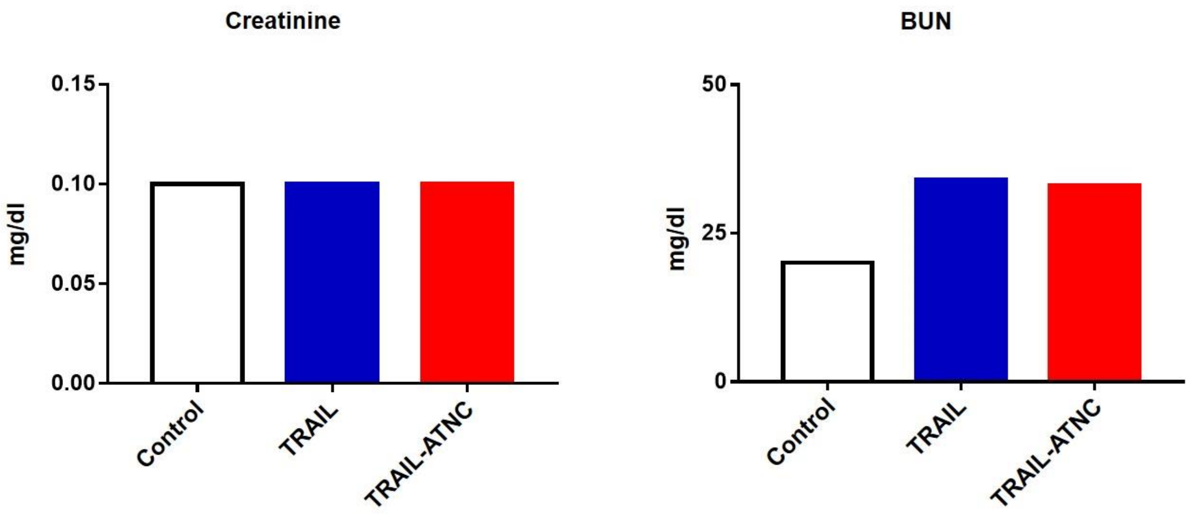

Figure S14. Hepatotoxicity and nephrotoxicity of TRAIL and TRAIL-ATNCs.

TRAIL or TRAIL-ATNC treated mice serum were tested for the hepatotoxicity and nephrotoxicity. (A) ALT and AST level of TRAIL or TRAIL-ATNC^{IL4rP} treated mice serum were within normal range. (B) Creatinine level of TRAIL or TRAIL-ATNC treated mice serum was same with normal mice. BUN level of TRAIL or TRAIL-ATNC treated mice serum was slightly higher than that of the normal mice but the levels are within the normal range.

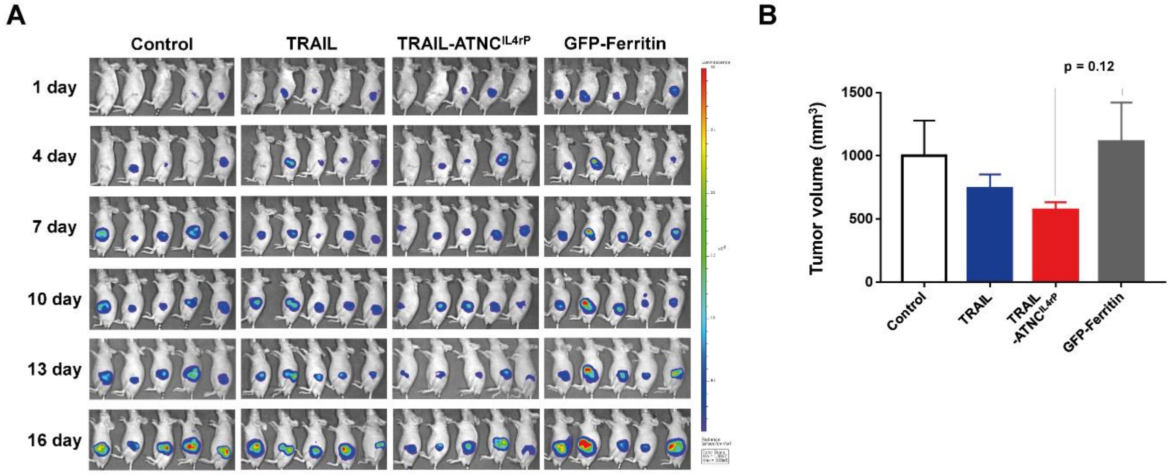

Figure S15. Bioluminescence imaging of the pancreatic cancer bearing mice under treatment and post-treatment tumor volumes.

(A) Sequential BLI imaging of mice of each treatment are shown demonstrating the increase in luciferase readout in control group mice and the significantly decreased readouts in TRAIL-ATNC^{IL4rP} treated mice. (B) Tumor volumes of each groups after anti-tumor therapy. Data represent means ± SEM (p = 0.12; T-test).
